# Free electron interaction with genistein: positive and negative ion formation

**DOI:** 10.1039/d5ra05594f

**Published:** 2025-11-26

**Authors:** Vy T. T. Nguyen, Jiakuan Chen, Milan Ončák, Stephan Denifl

**Affiliations:** a Institut für Ionenphysik und Angewandte Physik, Universität Innsbruck Technikerstrasse 25 A-6020 Innsbruck Austria milan.oncak@uibk.ac.at stephan.denifl@uibk.ac.at; b Center for Molecular Biosciences Innsbruck, Universität Innsbruck Technikerstrasse 25 A-6020 Innsbruck Austria

## Abstract

Genistein is a member of the group of isoflavones, which are present in edible plants and possess several health supporting properties. In this work we used a crossed beam experiment coupled to mass spectrometry. We investigated the formation of anions and cations from neutral genistein upon the interaction with electrons having kinetic energies from about 0 eV to 70 eV. In the case of negative ion formation, we find the intact negatively charged genistein as the most abundant anionic species. The dehydrogenated parent anion is observed as the only fragment anion formed by dissociative electron attachment to genistein. The parent and dehydrogenated species also represent prominent cations. However, we also observe abundant signals for ions formed upon cleavage of the centred ring *via* retro-Diels–Alder rearrangement. Quantum chemical calculations on the threshold energies support the experimentally found appearance energies.

## Abstract 2

In the present study we show that the isoflavone genistein has a pronounced molecular stability towards low-energy electron attachment while ionizing electrons efficiently induces retro-Diels–Alder rearrangement in the molecule.

## Graphical abstract

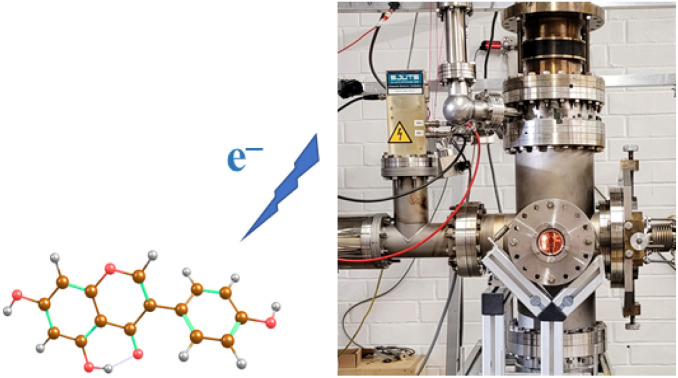

## 1 Introduction

**Fig. 1:**
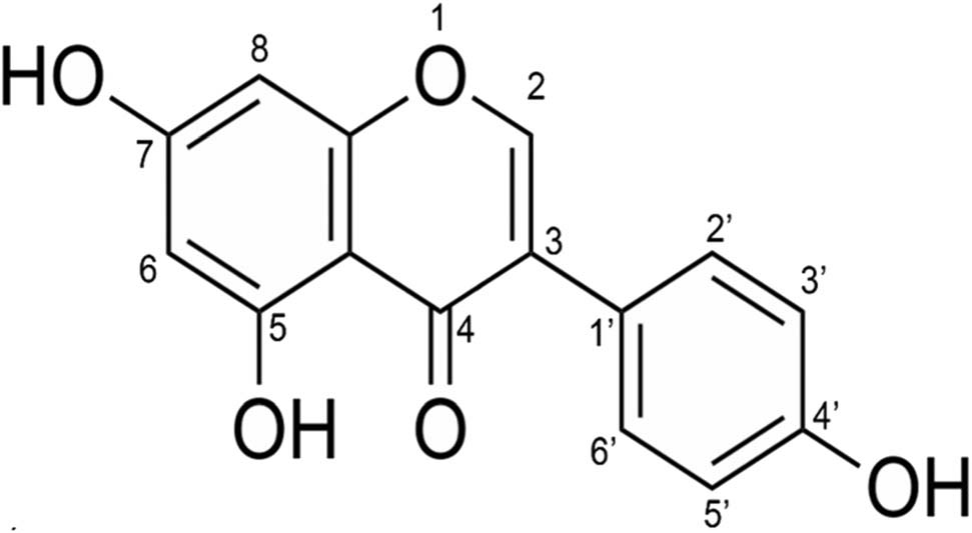
Molecular structure of genistein.

Genistein (C_15_H_10_O_5_, see Fig. 1 for the molecular structure) is an important isoflavone and can be found in many edible plants. It is one of the most predominant isoflavones consumed in daily diets.^1^ Genistein has been determined to bring about many health benefits and thus plays a central role in the prevention of various dysfunctions. It is reported that genistein has protective effects against cardiovascular diseases by lowering cholesterol levels.^2,3^ This isoflavone is also well known as a phytoestrogen that functions like the hormone estrogen in mammals. The potential of genistein in prevention of osteoporosis, a chronic disease, has been recently reported.^4,5^

Additionally, genistein exhibits many other physiological properties which are associated with cancer treatment and prevention including anti-inflammatory, antiproliferative, and anti-angiogenesis.^6^ Therefore, this compound has also been reviewed as an anti-cancer agent against various types of cancers. Action of genistein in hormone-related cancers such as breast cancers or prostate cancers has been extensively examined,^7^ and this compound has been found to exert anticancer mechanisms in brain tumours, gastrointestinal cancers, lung, bone as well as skin cancers. Genistein functions as a chemotherapeutic drug^8^ mainly *via* promoting apoptosis, altering the cell cycle and inhibiting metastasis.^8^ On the other hand, genistein has been recently reported as one of promising natural radiosensitisers^9^ in radiotherapy, a modality of oncology treatment, which employs ionising radiation to alleviate and cure tumours.^10–13^

Electron collisions play an important role in various phenomena in atmospheric physics, plasma applications, as well as astrochemical physics, and thus have been extensively investigated for recent decades with different motivations. In radiation physics and chemistry low-energy electron reactions with DNA constituents have received increased attention since Sanche and co-workers^14,15^ demonstrated strand break formation in dry DNA upon electron attachment (EA). EA studies with gas-phase nucleobases such as cytosine, thymine,^16,17^ uracil,^18^ adenine^19,20^ were carried out and complemented by studies with sugar units^21,22^ and phosphate groups.^23^ Subsequently the investigations were extended towards more complex molecular systems including nucleosides^24,25^ and nucleotides.^26,27^ On the other hand, biologically relevant molecules^20,28,29^ have also been studied towards electron ionisation (EI). EA and EI are used to explain many phenomena in radiation damage. Thus, electron collision studies were devoted to the clinically applied radiosensitiser nimorazole^30^ or promising radiosensitiser molecules^31–34^ in order to better understand their sensitising mechanisms in radiation therapy, which is still an open question in the state-of-the-art research.

In this work, we investigated interactions of low-energy electrons with genistein molecules in the gas phase upon elementary EA and EI. The study concept allows a better understanding of basic chemical–physical properties of genistein. Concerning negative ion formation upon EA, we found two relevant anion species with the parent anion as predominant species. Thermodynamics of observed anions were estimated by quantum chemical calculations. The EI mass spectrum of genistein molecules indicated several open dissociation channels at the electron energy of 70 eV. For prominent cations found in the mass spectrum, we determined their threshold energies. These onset values were also compared with those predicted by quantum chemical calculations.

## 2 Methods

### 2.1 Experimental methods

Experiments were performed with a crossed beam setup consisting of a high-resolution electron monochromator, a resistively heated oven, a quadrupole mass spectrometer and a detector for the ions.^35^ A nearly monoenergetic electron beam was generated with the hemispherical electron monochromator. The neutral molecular beam was produced by sublimating the solid sample filled in the heated oven in order to transfer the studied molecules into the gas phase. Gas-phase molecules were introduced as an effusive beam *via* a capillary of 1 mm diameter into the interaction area where the beam met the well-defined electron beam. Positive and negative ions may form upon EI and EA, respectively. The generated ions were then extracted towards the quadrupole mass spectrometer with a weak electrostatic field, mass analysed, and finally detected with a channeltron type secondary electron multiplier. In order to form a sufficiently intense molecular beam for collision experiments, the genistein sample (TCI Germany, stated purity of 98%, used as delivered) was refilled once and heated up to 442 K, measured with a PT100 sensor mounted directly on the copper oven. Whilst the current of electrons was around 20–50 nA monitored by a Faraday cup pico-ammeter, the working pressure was maintained at around 10^−7^ mbar during the measurements.

EA experiments with isolated genistein molecules in the gas phase were being conducted for multiple weeks. Measurements were repeated several times to confirm reproducibility and statistical significance. The electron energy scale was calibrated using the well-known Cl^−^ peak upon s-wave EA to CCl_4_ at 0 eV.^36^ The energy resolution of the electron beam was determined to be approximately 130 meV, defined as the full width at half maximum of the Cl^−^ Gaussian peak. On the other hand, the standard deviation of the calibrated Cl^−^ peak of 60 meV is considered as a systematic error. In our investigation, EA experiments were examined in the electron energy range 0–12 eV.

The EI mass spectrum of genistein was recorded at 70 eV incident electron energy. Subsequently, we carried out energy scans near the thresholds for five prominent cations and three different datasets were obtained. The ionisation energy of genistein as well as appearance energies of fragment cations of interest were determined by fitting a modified Wigner–Wannier function to the measured ion efficiency curves. The fitting procedure was already employed in previous studies.^20,28,29^ The fitting function is given as:

1 *f*(*E*) = *b* + *c*·(*E* − AE)^*n*^·*θ*(*E* − AE)

The equation includes a Heaviside function *θ*. Therein, *E* represents the electron energy, AE is the experimental appearance energy and *n* is the exponential factor of the fitted function. *b* is a constant describing background contribution and *c* is the scaling factor of the fitting function. The electron energy scale was calibrated using the well-known Ar^+^ threshold energy at 15.763 eV.^37^

### 2.2 Computational methods

We performed quantum chemical calculations to explore structure and energetics of genistein and its fragments. For optimisation, we used the density functional theory (DFT) employing the *ω*B97XD functional^38^ along with the aug-cc-pVDZ basis set. In the optimized structures, a single-point *ω*B97XD/aug-cc-pVTZ calculation was performed to obtain more reliable energetics, employing the zero-point energy as calculated at the *ω*B97XD/aug-cc-pVDZ level. Wave function stabilisation was performed prior to every calculation. Several isomers were located for larger molecules, their structures are shown in the SI, Fig. S1–S6. All calculated molecules represent local minima on the potential energy surface. Gaussian software was used for all calculations.^39^

## 3. Results and discussion

### 3.1 Electron attachment

In the present study, we investigated EA to genistein molecules under isolated conditions in the 0–12 eV electron energy range. The observed variety of anionic reaction products formed from EA to genistein turned out to be very limited. Only two different anionic species were observed within the detection limit of the presently used apparatus. One of both was the intact genistein parent anion at *m*/*z* (mass-to-charge ratio) 270. The other species was the dehydrogenated genistein anion at *m*/*z* 269, [G–H]^−^, which is formed upon the removal of a hydrogen atom from the molecule:

2 e^−^ + G → (G^−^)^#^ → G^−^

3 e^−^ + G → (G^−^)^#^ → [G–H]^−^ + H

(G^−^)^#^ represents the transient negative ion (TNI) of genistein.

**Fig. 2:**
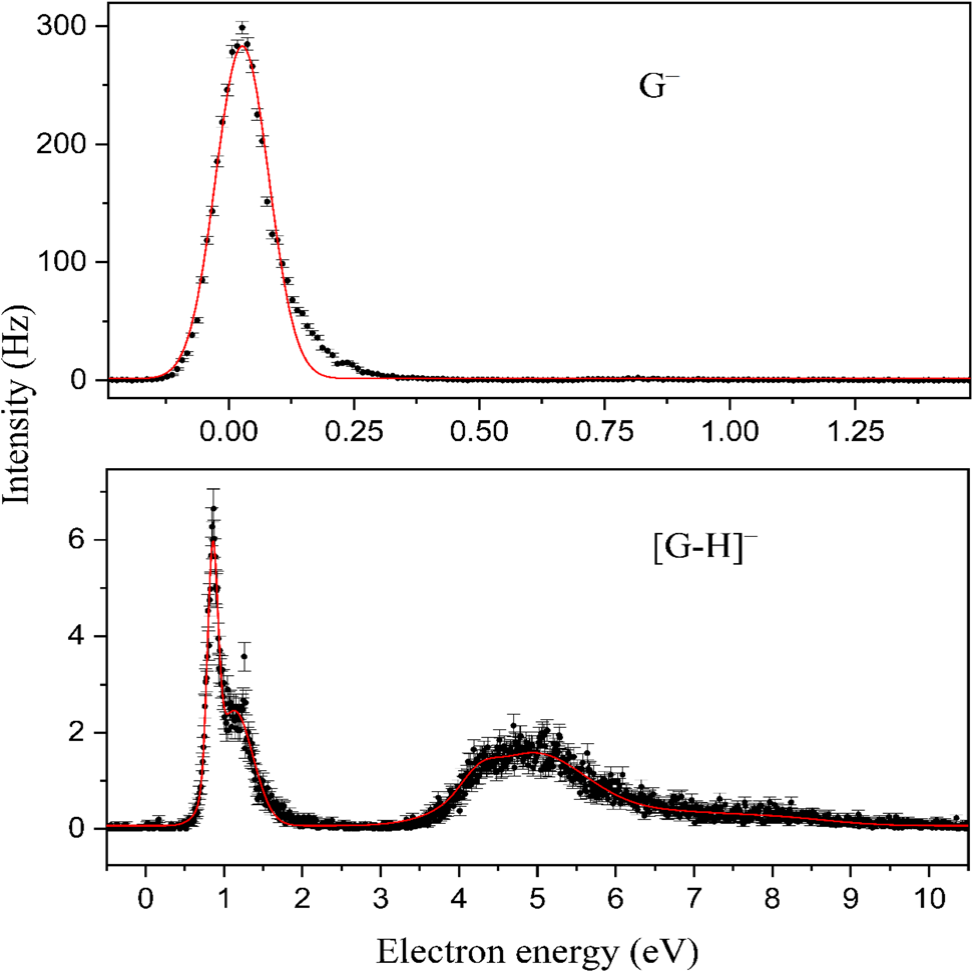
Anion efficiency curves for the formation of genistein parent anion at *m*/*z* 270 and that of dehydrogenated genistein anion at *m*/*z* 269, the only observable fragment. The black dots represent the experimental data with error bars whereas the fitting with Gaussian functions is given by the solid red line. Measurements were repeated several times. The error bars refer to the standard deviation of the mean, calculated by 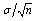 with *σ* is the standard deviation of the mean value and *n* is the total number of measurements.

**Fig. 3:**
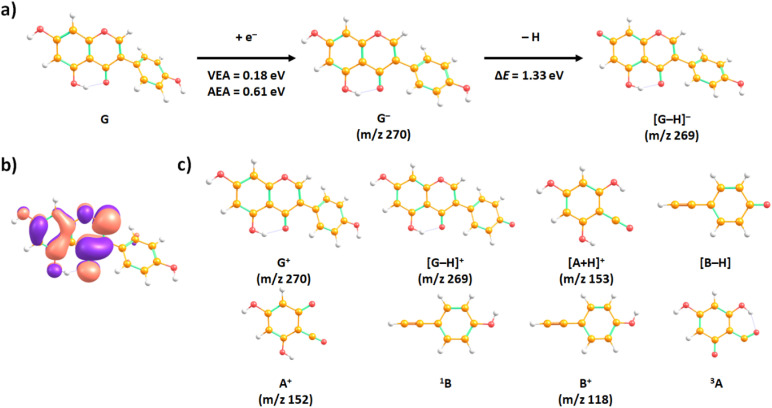
(a) Suggested reaction pathway upon electron attachment to genistein (G), along with vertical and adiabatic electron affinities (VEA and AEA, respectively), and the reaction energies Δ*E* for abstraction of a neutral hydrogen atom. (b) The singly occupied orbital upon electron attachment to genistein in the lowest electronic state. (c) Cations and neutrals formed upon ionisation of genistein. All results were obtained at the *ω*B97XD/aug-cc-pVTZ//*ω*B97XD/aug-cc-pVDZ level of theory. Numbers in superscript denote spin multiplicity.

**Fig. 4:**
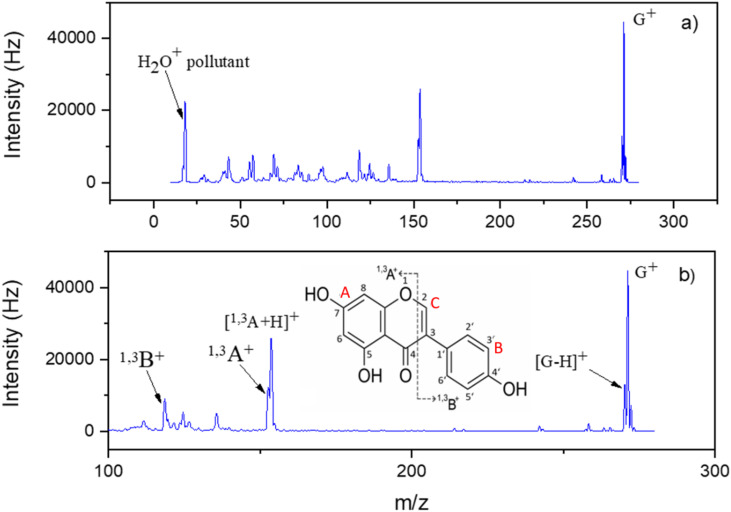
Electron ionisation spectrum of genistein recorded at 70 eV electron energy. (a) Entire mass spectrum with *m*/*z* range of 0–300. The peak at *m*/*z* 18 is a pollutant (water), originating from the machine background. (b) Enlarged spectrum with *m*/*z* above 100. The most prominent cations resulting from electron ionisation of genistein molecules are assigned, following a nomenclature from collision induced dissociation.^63^ Therefore, the rings are labelled with A, B and C (shown in red). The ^1,3^A^+^ and ^1,3^B^+^ labels represent cations containing intact A and B rings, respectively, see molecular structure in (b). The superscripts 1 and 3 indicate the position of the specific bond cleavages in the ring labelled with C.^64^

Fig. 2 shows the anion efficiency curves of the aforementioned negative ions as a function of incident electron energy. Table 1 summarizes the information on the peak positions of the anions observed, which was derived by peak fitting. As can be seen in Fig. 2, the formation of the intact genistein anion takes place within a narrow peak nearly at 0 eV. Our calculations suggest that the formation of the transient genistein anion is an exothermic process, *i.e.*, a bound state is formed (Fig. 3a). We obtain +0.18 eV for the vertical electron affinity and +0.61 eV for the adiabatic electron affinity of the most stable genistein isomer found. Upon EA, the odd electron resides in an π orbital of ring A (see Fig. 4 for the nomenclature) and the structure changes only slightly. The main structural change is related to the lowering of the angle between the two ring moieties from ∼45° to ∼25° (Fig. 3a and b).

**Table 1:** Anions generated by (dissociative) electron attachment to isolated genistein in the gas phase, together with their resonance peak energies. The systematic error of 60 meV resulting from the calibration of the electron energy scale is applied to all experimentally determined peak positions. Calculated threshold energies are provided at the *ω*B97XD/aug-cc-pVTZ//*ω*B97XD/aug-cc-pVDZ level

Mass (u)	Anion species	Peak position (eV)						Calculated threshold (eV)
270	G^−^	0.0						−0.61
269	[G–H]^−^	0.8	1.1	4.2	4.9	6.7	8.0	0.72

A positive electron affinity of genistein may imply an extension of the TNI lifetime towards the microsecond timescale, which is required for detection by mass spectrometry. For comparison, the time required for detection of the genistein anion in the present experiment is about 300 µs. Capturing an electron with kinetic energy of nearly zero eV means that just the electron affinity is released as vibrational excess energy in the ground state TNI (vibrational Feshbach resonance). Intramolecular vibrational redistribution (IVR) may lower the vibrational excess energy in the individual reaction coordinates and prevents temporarily spontaneous autodetachment. Such effective process was previously suggested for the SF_6_^−^ parent anion.^40^ However, in the current experiment, no experimental means exist to confirm the latter process and it should be noted that the electron affinity of SF_6_ is significantly higher (1.06 eV^41^), *i.e.* more excess energy must be distributed in the latter case.

At electron energies above zero eV, we observe dehydrogenated genistein anion, which is the only fragment anion observed within the detection limit of the present apparatus. Previously, the dehydrogenation process upon EA was observed as most abundant reaction channel for several molecules of biomolecular relevance.^24,42–44^ As described by reaction (3), the dehydrogenated genistein anion is generated simultaneously with a neutral hydrogen radical. The anion efficiency curve of [G–H]^−^ is presented in the lower panel of Fig. 2. It shows rather narrow peaks close to about 1 eV and broad features between about 4 and 10 eV. The formation over such wide range of electron energies upon different resonances may also be associated with H abstraction from different sites of the molecule. The lowest predicted thermodynamic energy threshold is 0.72 eV, which is associated with the H-elimination from the hydroxyl group at C7 position (see Fig. 3a). This pathway is most probably responsible for the peak at ∼0.8 eV in the [G–H]^−^ anion efficiency curve. The peak threshold of ∼0.6 eV is in good agreement with the calculations. Such site selectivity upon dissociative electron attachment (DEA) was previously suggested for small organic molecules^45,46^ as well as cyclic molecules like nucleobases.^47–49^ A possible experimental approach to study this chemical selectivity by mass spectrometry involved the study of partially deuterated molecules.^45,49^

The second peak in the [G–H]^−^ anion efficiency curve is observed at 1.1 eV. It may be also formed again by the H removal from hydroxyl group at C7 position of the ring A or by H abstraction from the 4′ site of ring B with a computational threshold of 1.15 eV (see iso3 and iso4 of [G–H]^−^ in Fig. S2). This calculated threshold is thus close to the position of the peak maximum. Further isomers of [G–H]^−^ with H abstraction from the hydroxyl group at C5 position and from carbon atoms, iso5–13 in Fig. S2, have calculated thresholds of 1.44–3.38 eV. However, no discernible features are present around these energies (tail of second peak), and thus we do not expect that they contribute to [G–H]^−^ formation at lower electron energies. The situation may be different for the ion yield above about 4 eV, which may be formed upon core-excitation, *i.e.* an electron of the molecule is promoted to a formerly unfilled orbital upon resonance formation.^50^

The present results indicate a remarkable stability of genistein towards DEA. The fragment anion [G–H]^−^ is generated with only about 5% relative intensity to the intact parent anion (derived from the peak maxima). Genistein shares this property of abundant parent anion formation with low DEA efficiency with *para*-benzoquinone,^51,52^ and certain derivative of it,^53–55^ polyaromatic hydrocarbons^56^ and the well-known highly stable fullerenes.^57^ The dissociation process may involve initial formation of a π* resonance with subsequent coupling to a dissociative σ*(OH orbital). Another possibility may include a direct attachment of the excess electron into the σ*(OH) orbital, which is favoured for example for carboxylic group from amino acids.^58^ Within the detection limit of the apparatus, we do not observe abstraction of the neutral hydroxyl radical as well as the negatively charged hydroxyl, though OH has a considerable electron affinity of about 1.83 eV.^59^ Previously the release of OH was suggested for the bicyclic tirapazamine molecule as a result of roaming process.^31^ Modelli and Pshenichnyuk studied EA to three flavonoids (naringenin, quercetin and myricetin) and observed that just myricetin with its six hydroxyl groups undergoes OH release.^60^ However, this dissociation channel turned out to be rather weak with the corresponding anion having 0.3% relative intensity compared to the parent anion. Since it can be assumed that genistein has a variety of π* resonances like other flavonoids investigated in ref. 60, which may act as doorway state to DEA, the limited dissociation may be a result of efficient IVR in the genistein transient negative ion. In contrast, DEA to 2,3-dimethoxy-5-methylhydroquinone (CoQ_0_H_2_) in the gas phase led to intense formation of [CoQ_0_H_2_–OH]^−^ in a resonance near 1.6 eV. OH^−^ was just formed at higher electron energies near 7 eV and had about a factor 100 lower intensity than [CoQ_0_H_2_–OH]^−^.^61^ The structurally related hydroquinone group has thus a considerable higher reactivity towards electron induced dissociation than genistein. We just note that the benzoquinone unit was considered as electrophore within the electron transport chains of respiration and photosynthesis.^62^

### 3.2 Electron ionisation

Fig. 4 shows the measured EI mass spectrum of genistein at the incident electron energy of 70 eV. The mass spectrum indicates formation of several fragment cations, though the most intense peak at *m*/*z* 270 can be assigned to the genistein parent cation G^+^. The four most abundant fragment cations are discernible in the enlarged ionisation spectrum, Fig. 4b. Their *m*/*z* are all above 100. Using a nomenclature from mass spectrometry with (iso)flavones,^63,64^ these mass peaks in the spectrum are assigned to ^1,3^B^+^ (*m*/*z* 118, C_8_H_6_O^+^), ^1,3^A^+^ (*m*/*z* 152, C_7_H_4_O_4_^+^), [^1,3^A + H]^+^ (*m*/*z* 153, C_7_H_5_O_4_^+^) and [G–H]^+^ (*m*/*z* 269) fragment cations, see also Table 2, which lists the relative intensities to the parent cations (the superscript next to A and B denotes the position where the ring is broken). We just note that the present mass spectrum closely reproduces the EI mass spectrum of genistein in the Spectral Database for Organic compounds (SDBS).^65^

**Table 2:** List of cations from genistein together with the assigned structure, relative intensity in the mass spectrum, experimental appearance energies and calculated thresholds. Uncertainties are related to the error resulting from the fitting. Calculated threshold energies are provided at the *ω*B97XD/aug-cc-pVTZ//*ω*B97XD/aug-cc-pVDZ level

Mass (u)	Assigned cations	Relative intensity (%)	Appearance energy (eV)		Theoretical energy threshold (eV)
270	G^+^	100	7.95 ± 0.02	—	7.6
269	[G–H]^+^	28.6	11.44 ± 0.14	12.32 ± 0.05	11.2
153	[^1,3^A + H]^+^	57.5	11.48 ± 0.25	12.31 ± 0.16	11.1
152	^1,3^A^+^	27.5	11.05 ± 0.19	12.89 ± 0.16	11.2
118	^1,3^B^+^	19.9	12.98 ± 0.24	15.13 ± 0.11	12.8

**Fig. 5:**
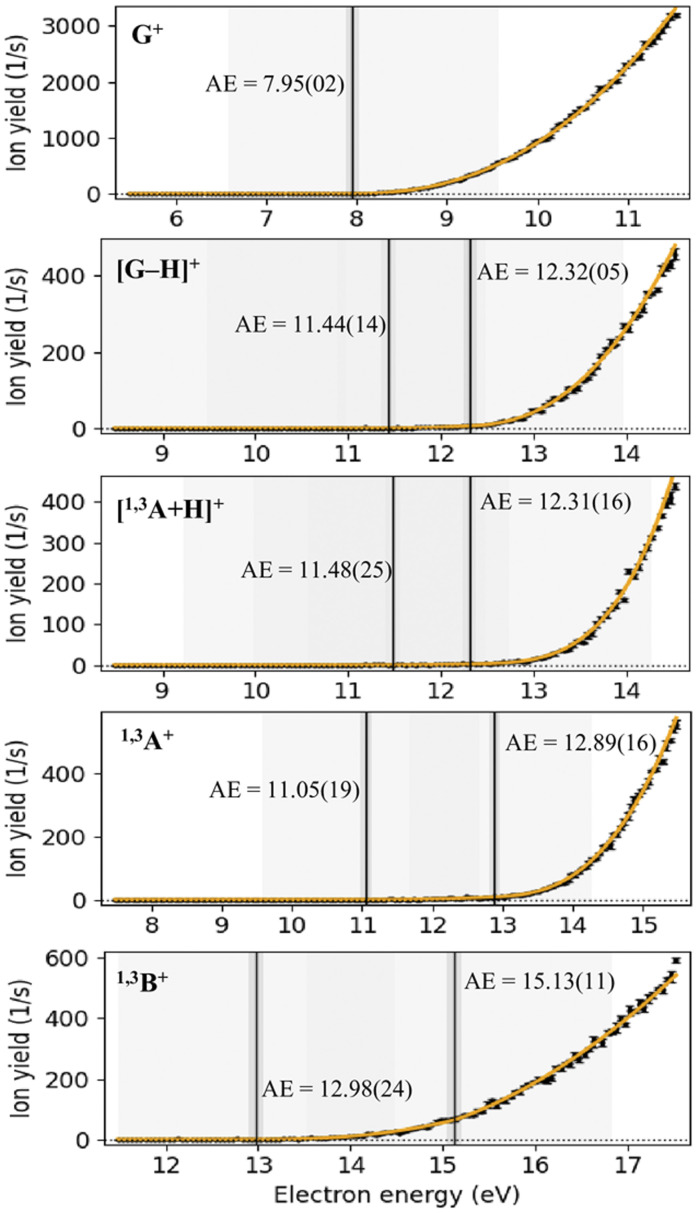
Ion efficiency curves for prominent cations formed upon EI of genistein. Experimental data are illustrated as black dots with error bars calculated as the standard deviation of the mean, whereas the solid orange line represents the corresponding fitting function. The energy thresholds derived by the applied fitting method are indicated as the straight black lines together with the corresponding numerical values.

We investigated the onset of ion formation for G^+^, [G–H]^+^, [^1,3^A + H]^+^, ^1,3^A^+^, and ^1,3^B^+^ by conducting energy scans close to the threshold region. The corresponding measured ion efficiency curves as a function of incident electron energy are depicted in Fig. 5. Applying the modified Wigner–Wannier fitting function mentioned in the experimental methods section, we derived the corresponding AEs from these scans. The obtained AE values are summarised together with the uncertainties resulting from the fitting procedure in Table 2. The exponential factors of the fitted Wigner–Wannier functions are listed in the SI (Table S1).

To support the experimental results, we have also determined the theoretical adiabatic ionisation energy for the parent cation and AEs for the other four prominent cations, as listed in Table 2. Fig. 3c illustrates calculated optimal structures of the fragments resulting from EI of the genistein molecule in the gas phase.

The most intense peak in the mass spectrum can be assigned to the genistein parent cation G^+^,

4 e^−^ + G → G^+^ + 2e^−^

The experimental AE of genistein is found at 7.95 eV (Fig. 5), whereas the calculated adiabatic ionisation energy is determined as 7.59 eV. For comparison, Lengyel *et al.* derived a similar ionisation energy (IE) of 7.45 eV in DFT calculations.^66^ A recent DFT work predicted an IE of 7.30 eV.^67^ Thus, the measured AE of genistein parent cation G^+^ is slightly higher than the calculated one, which indicates that the ground state of molecular cation is not directly reached upon the ionisation process. We also calculated the vertical ionisation energy and obtained a value of 7.91 eV, matching well with the experimental value. As expected, the AE of the intact genistein cation is experimentally and theoretically the lowest value of all cations under investigation. We also note that Lengyel *et al.* calculated the IE for a series of other isoflavones.^66^ It turned out that the IE values of the ten investigated molecules were rather similar, ranging from 7.24 eV (sanatal) to 7.44 (daidzein).^66^ For comparison, Kobayashi *et al.* carried out photoelectron spectroscopy experiments with *trans*- and *cis*-stilbene and reported vertical IE values of 7.87 eV and 8.17 eV, respectively.^68^ Few IE values for stilbene obtained upon photon impact and charge transfer can be also found on the NIST chemistry homepage.^69^ Most of these IEs range between about 7.5 eV and 8.0 eV and are thus similar to that of genistein.

For other prominent cations, as can be seen in Fig. 5, there are two experimental thresholds apparent in the cation efficiency curves. The second thresholds are more pronounced than the first ones, except for the ^1,3^B^+^ ion efficiency curve. The same trend appears for the AEs, while the experimental AEs of ^1,3^A^+^, [^1,3^A + H]^+^, [G–H]^+^ are slightly close to each other (first measured thresholds are around 11.05–11.48 eV, whereas those for the second onsets are in the range of 12.31–12.89 eV), the two experimental energy thresholds for the cation ^1,3^B^+^ are by far the highest values observed.

The calculated AEs exhibit good agreement with the measured values for all prominent fragments, like for example the dehydrogenated genistein cation at *m*/*z* 269. This fragment cation could be emitted *via* the following reaction:

5 e^−^ + G → [G–H]^+^ + H + 2e^−^

As mentioned before, there are two experimental energy onsets for the appearance of this fragment. The first one is found at 11.44 eV, which is near to the corresponding theoretical value predicted at 11.2 eV for removal of H from the OH group on 4′ position (Fig. 3c). For an elimination of a neutral hydrogen atom from the hydroxyl group at position 7, by 0.60 eV more energy is needed, although this isomer is energetically most favourable for the formation of the dehydrogenated parent anion [G–H]^−^ (see Table 2 and Fig. S3). The second energy threshold of dehydrogenated genistein cation was experimentally determined at 12.32 eV, which could be explained *via* the H abstraction at different positions (see Fig. S3).

Another fragment cation, which is produced with a nearly similar relative intensity as the dehydrogenated genistein cation [G–H]^+^, is the ^1,3^A^+^ cation, formed in the ionisation reaction,

6 e^−^ + G → ^1,3^A^+^ + ^1,3^B + 2e^−^

The counterpart fragment ^1,3^B^+^ can be formed *via* the suggested reaction below,

7 e^−^ + G → ^1,3^A + ^1,3^B^+^ + 2e^−^

The mass spectrum indicates that both fragments are generated with slightly different probabilities, with 27.5% and 19.9%, resulting in the peaks at *m*/*z* 152 and 118, respectively.

**Fig. 6:**
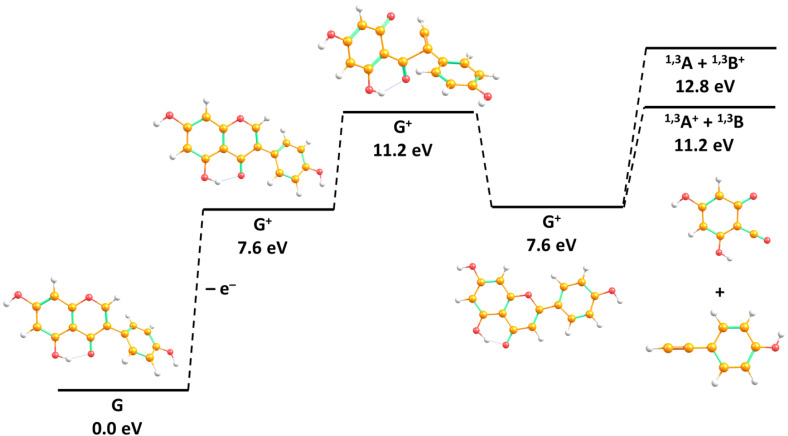
Simplified reaction path for formation of ^1,3^A^+^ + ^1,3^B and ^1,3^A + ^1,3^B^+^ from G as calculated at the *ω*B97XD/aug-cc-pVTZ//*ω*B97XD/aug-cc-pVDZ level of theory. See Fig. S7 for the complete pathway.

^1,3^A^+^ and ^1,3^B^+^ are fragments formed from the retro-Diels–Alder (rDA) rearrangement^70–72^ on the pyran ring (denoted with C) of the genistein molecular structure, as indicated in the Fig. 4b. Our calculated reaction pathway for the rDA reaction (Fig. 6 and S7) show that no barrier on the potential energy surface lies above the energy of the dissociation channel to form ^1,3^A^+^ or ^1,3^B^+^.

In the simplest possible system for a rDA reaction upon EI ionisation, the cyclohexene cation breaks up into either ionised ethylene or ionised butadiene, as observed by Budzikiewicz *et al.*^73^ The rDA dissociation pathway has been reported in previous mass spectrometric studies on genistein and other isoflavones and it was also suggested that this reaction could be used in analytical chemistry for identification of unknown isoflavones.^71^ For example, Nakata *et al.*^64^ has investigated the (argon) collision induced dissociation (CID) of protonated genistein formed by electrospray ionisation method and observed (protonated) ^1,3^A^+^ as the most abundant fragment. For CID of the deprotonated genistein in negative electrospray ionisation, product ions resulting from the retro-cyclisation cleavage were observed with a very small proportion.^74^ The SDBS also includes EI mass spectra for two flavone derivatives, 5,7-dihydroxyflavone and 5,7,3′,4′-tetrahydroxyflavone, respectively.^65^ EI of the 5,7-dihydroxyflavone molecule at 70 eV leads to the ^1,3^A^+^ as most abundant fragment ion.^65^ The EI mass spectrum of flavone which is included in the NIST database also shows abundant formation of ^1,3^A^+^ by rDA reaction.^69^ In the case of 5,7,3′,4′-tetrahydroxyflavone, another cleavage reaction of the centred ring seems abundantly to occur as the two most abundant fragment ions indicate in the mass spectrum. All reported EI spectra of these flavones further indicate the efficient loss of small neutral fragments from the parent cation, like hydrogen and carbon monoxide. While here we find abundant H-loss in EI of genistein as well, the loss of neutral CO represents a minor channel in the mass spectrum shown in Fig. 4. It is interesting to note that the EI spectra of *trans*- and *cis*-stilbene indicate loss of hydrogen atoms and methyl group as the major fragmentation reactions of the parent cations.^69^

In the present experiments, the first energy onset for cation ^1,3^A^+^ is observed at 11.05 eV whereas that of theoretical one is slightly above, predicted at 11.2 eV. The second experimental AE is measured at 12.89 eV. This onset is possibly associated with one/more higher lying transition state/s in between, *i.e.* the ionic state cannot be accessed directly from the neutral precursor. A similar phenomenon is also observed for the complementary fragment ^1,3^B^+^, however, requiring more energy. In this case, the first experimental AE was found at 12.98 eV, with the calculated value of 12.8 eV. The second onset was experimentally observed at 15.13 eV. The formation of ^1,3^B^+^ above this threshold could be possible from other fragmentation mechanisms, which require more transition states to be reached. The higher AEs of ^1,3^B^+^ compared to the ^1,3^A^+^ fragment cation may also explain the overall lower abundance of ^1,3^B^+^ than ^1,3^A^+^ in the mass spectrum shown in Fig. 4. We note that the calculated AE of B^+^ might drop to 11.8 eV if the most stable A isomer with deprotonated OH group at position 7 is used (see iso4 of singlet ^1,3^A in Fig. S6). However, the considerable mismatch between the observed and calculated values suggests that this isomer is not favoured kinetically due to the need for a proton transfer.

The most intense peak after the parent cation in the mass spectrum (accounted for 57.5% relative intensity) can be assigned to formation of [^1,3^A + H]^+^. The following reaction may be considered,

8 e^−^ + G → [^1,3^A + H]^+^ + [^1,3^B–H] + 2e^−^

As indicated in Fig. 4, the first threshold energy for the [^1,3^A + H]^+^ formation was experimentally found to be 11.48 eV, somewhat above the theoretical value at 11.1 eV, see Table 2. The structure of [^1,3^A + H]^+^ shown in Fig. 3c suggests that the additional proton is bound to the O1 oxygen. The second onset energy was measured at 12.31 eV, which may be also associated with transition states leading to different dissociation mechanisms from the suggested reaction (8). The observed high efficiency of the proton transfer within the rDA reaction is in line with the results of collision induced dissociation studies by Nakata *et al.*^64^ Using genistein molecule with deuterated B and C rings, they obtained [^1,3^A + D]^+^ as the major product ion in CID of the molecule initially protonated during the electrospray ionisation process to transfer the sample into the gas phase. We further note that the missing formation of [^1,3^A + H]^+^ in the SDBS mass spectrum for 5,7-dihydroxyflavone^65^ may be explained the absence of the OH group at the B ring, which acts as the proton source in the case of genistein (see Fig. 3).

## 4. Conclusions

In the present study, we provide a detailed view on the interaction of electrons with kinetic energies ≤70 eV and the genistein molecule in the gas phase. We observed a high stability of the molecule towards dissociation. Irrespective if negative ions are formed below the electron energy of 10 eV or cation formation above a minimum electron energy of 7.95 eV occurs, the charged intact genistein compound is formed predominantly. Another similarity between electron attachment and electron ionisation in genistein are related to the dehydrogenation reaction, which is abundantly observed for both opposite charge states. A major difference appears for other dissociation reactions, since fragment cations formed upon ring cleavage with rDA rearrangement are abundant, while genistein resonances do not lead to this dissociation (or the intensity of associated anions formed is below detection limit of apparatus). Previously, Bowie and Ho observed rDA reactions in EA to bicyclic compounds.^75^ However, they pointed out two basic conditions to observe the reaction in negative ions, (i) heterocyclic 1,3 or 1,4 dioxins are more favourable against cyclohexene and (ii) and the presence of nitrogen dioxide group in the molecule. While the former promotes the corresponding cleavage of the ring, the NO_2_ group may promote the electron capture leading to considerable DEA cross sections.^76^ Indeed, the overall anion yields observed presently are rather low compared to those from an electron scavenger like studied in ref. 76 and compared to the yields for positive formation. We also note that a high biological relevance in terms of antioxidant activity of flavonoids was suggested for a possible double dehydrogenation reaction by solvated electrons.^60^ Here we just observed emission of a single reactive H radical in the interaction of free low-energy electron with genistein.

## Author contributions

V. T. T. N. and J. C. performed the measurements, V. T. T. N. analysed the data, M. O. performed the quantum chemical calculations. V. T. T. N., M. O. and S. D. prepared the manuscript draft.

## Conflicts of interest

There are no conflicts to declare.

## Supplementary Material

RA-015-D5RA05594F-s001

## Data Availability

The data that support the findings of this study are available within the article and its supplementary information (SI). This manuscript has associated data in a data repository, see https://doi.org/10.5281/zenodo.17652744. Supplementary information is available. See DOI: https://doi.org/10.1039/d5ra05594f.

## References

[cit1] Yang Z., Kulkarni K., Zhu W., Hu M. (2012). Anti-Cancer Agents Med. Chem..

[cit2] Sirtori C. R., Lovati M. R. (2001). Curr. Atheroscler. Rep..

[cit3] Si H., Liu D. (2007). Curr. Med. Chem..

[cit4] Anderson J. J., Garner S. C. (1998). Baillieres Clin. Endocrinol. Metab..

[cit5] Messina M., Ho S., Alekel D. L. (2004). Curr. Opin. Clin. Nutr. Metab. Care.

[cit6] Sharifi-Rad J., Quispe C., Imran M., Rauf A., Nadeem M., Gondal T. A., Ahmad B., Atif M., Mubarak M. S., Sytar O., Zhilina O. M., Garsiya E. R., Smeriglio A., Trombetta D., Pons D. G., Martorell M., Cardoso S. M., Razis A. F. A., Sunusi U., Kamal R. M., Rotariu L. S., Butnariu M., Docea A. O., Calina D. (2021). Oxid. Med. Cell. Longevity.

[cit7] Konstantinou E. K., Gioxari A., Dimitriou M., Panoutsopoulos G. I., Panagiotopoulos A. A. (2024). Int. J. Mol. Sci..

[cit8] Spagnuolo C., Russo G. L., Orhan I. E., Habtemariam S., Daglia M., Sureda A., Nabavi S. F., Devi K. P., Loizzo M. R., Tundis R., Nabavi S. M. (2015). Adv. Nutr..

[cit9] Komorowska D., Radzik T., Kalenik S., Rodacka A. (2022). Int. J. Mol. Sci..

[cit10] Raffoul J. J., Wang Y., Kucuk O., Forman J. D., Sarkar F. H., Hillman G. G. (2006). BMC Cancer.

[cit11] Tang Q., Ma J., Sun J., Yang L., Yang F., Zhang W., Li R., Wang L., Wang Y., Wang H. (2018). Oncol. Rep..

[cit12] Liu X., Sun C., Jin X., Li P., Ye F., Zhao T., Gong L., Li Q. (2013). Molecules.

[cit13] Shi Y. (2002). Mol. Cell.

[cit14] Boudaiffa B., Cloutier P., Hunting D., Huels M. A., Sanche L. (2000). Science.

[cit15] Huels M. A., Boudaïffa B., Cloutier P., Hunting D., Sanche L. (2003). J. Am. Chem. Soc..

[cit16] Denifl S., Ptasinska S., Cingel M., Matejcik S., Scheier P., Märk T. D. (2003). Chem. Phys. Lett..

[cit17] Denifl S., Ptasińska S., Probst M., Hrǔsak J., Scheier P., Märk T. D. (2004). J. Phys. Chem. A.

[cit18] Denifl S., Ptasińska S., Hanel G., Gstir B., Probst M., Scheier P., Märk T. D. (2004). J. Chem. Phys..

[cit19] Denifl S., Sulzer P., Huber D., Zappa F., Probst M., Märk T. D., Scheier P., Injan N., Limtrakul J., Abouaf R., Dunet H. (2007). Angew. Chem., Int. Ed..

[cit20] Dawley M. M., Tanzer K., Cantrell W. A., Plattner P., Brinkmann N. R., Scheier P., Denifl S., Ptasińska S. (2014). Phys. Chem. Chem. Phys..

[cit21] Bald I., Kopyra J., Illenberger E. (2006). Angew. Chem., Int. Ed..

[cit22] Ptasińska S., Denifl S., Scheier P., Märk T. D. (2004). J. Chem. Phys..

[cit23] König C., Kopyra J., Bald I., Illenberger E. (2006). Phys. Rev. Lett..

[cit24] Parida D., Chen J., Schorr L., Nguyen V. T. T., Saqib M., Bayer A., Zappa F., Denifl S. (2025). Eur. Phys. J. D.

[cit25] Muftakhov M. V., Shchukin P. V., Khatymov R. V. (2021). Radiat. Phys. Chem..

[cit26] Kopyra J. (2012). Phys. Chem. Chem. Phys..

[cit27] Chomicz-Mańka L., Czaja A., Falkiewicz K., Zdrowowicz M., Biernacki K., Demkowicz S., Izadi F., Arthur-Baidoo E., Denifl S., Zhu Z., Ahmet Tufekci B., Harris R., Bowen K. H., Rak J. (2023). J. Am. Chem. Soc..

[cit28] Meißner R., Feketeová L., Ribar A., Fink K., Limão-Vieira P., Denifl S. (2019). J. Am. Soc. Mass Spectrom..

[cit29] Meißner R., Feketeová L., Bayer A., Postler J., Limão-Vieira P., Denifl S. (2019). J. Mass Spectrom..

[cit30] Meißner R., Kočišek J., Feketeová L., Fedor J., Fárník M., Limão-Vieira P., Illenberger E., Denifl S. (2019). Nat. Comm..

[cit31] Arthur-Baidoo E., Ameixa J., Ziegler P., Da Ferreira Silva F., Ončák M., Denifl S. (2020). Angew. Chem., Int. Ed..

[cit32] Sedmidubská B., Kočišek J. (2024). Phys. Chem. Chem. Phys..

[cit33] Saqib M., Arthur-Baidoo E., Izadi F., Szczyrba A., Datta M., Demkowicz S., Rak J., Denifl S. (2023). J. Phys. Chem. Lett..

[cit34] Lochmann C., Luxford T. F. M., Makurat S., Pysanenko A., Kočišek J., Rak J., Denifl S. (2022). Pharmaceuticals.

[cit35] Saqib M., Izadi F., Isierhienrhien L. U., Ončák M., Denifl S. (2023). Phys. Chem. Chem. Phys..

[cit36] Pelc A. (2022). Acta Phys. Pol., A.

[cit37] Weitzel K.-M., Mähnert J., Penno M. (1994). Chem. Phys. Lett..

[cit38] Chai J.-D., Head-Gordon M. (2008). Phys. Chem. Chem. Phys..

[cit39] Frisch M. J. , Trucks G. W., Schlegel H. B., Scuseria G. E., Robb M. A., Cheeseman J. R., Scalmani G., Barone V., Petersson G. A., Nakatsuji H., Li X., Caricato M., Marenich A. V., Bloino J., Janesko B. G., Gomperts R., Mennucci B., Hratchian H. P., Ortiz J. V., Izmaylov A. F., Sonnenberg J. L., Williams-Young D., Ding F., Lipparini F., Egidi F., Goings J., Peng B., Petrone A., Henderson T., Ranasinghe D., Zakrzewski V. G., Gao J., Rega N., Zheng G., Liang W., Hada M., Ehara M., Toyota K., Fukuda R., Hasegawa J., Ishida M., Nakajima T., Honda Y., Kitao O., Nakai H., Vreven T., Throssell K., Montgomery Jr. J. A., Peralta J. E., Ogliaro F., Bearpark M. J., Heyd J. J., Brothers E. N., Kudin K. N., Staroverov V. N., Keith T. A., Kobayashi R., Normand J., Raghavachari K., Rendell A. P., Burant J. C., Iyengar S. S., Tomasi J., Cossi M., Millam J. M., Klene M., Adamo C., Cammi R., Ochterski J. W., Martin R. L., Morokuma K., Farkas O., Foresman J. B. and Fox D. J., Gaussian 16 Revision A.03, Gaussian Inc. Wallingford CT, 2016

[cit40] Gerchikov L. G., Gribakin G. F. (2008). Phys. Rev. A.

[cit41] Christophorou L. G., Olthoff J. K. (2001). Int. J. Mass Spectrom..

[cit42] Baccarelli I., Bald I., Gianturco F. A., Illenberger E., Kopyra J. (2011). Phys. Rep..

[cit43] Gorfinkiel J. D., Ptasinska S. (2017). J. Phys., B.

[cit44] Fabrikant I. I., Eden S., Mason N. J., Fedor J. (2017). Adv. At., Mol., Opt. Phys..

[cit45] Prabhudesai V. S., Kelkar A. H., Nandi D., Krishnakumar E. (2005). Phys. Rev. Lett..

[cit46] Orzol M., Martin I., Kocisek J., Dabkowska I., Langer J., Illenberger E. (2007). Phys. Chem. Chem. Phys..

[cit47] Das G., Prabhudesai V. S., Sajeev Y. (2025). Commun. Chem..

[cit48] Kawarai Y., Weber T., Azuma Y., Winstead C., McKoy V., Belkacem A., Slaughter D. S. (2014). J. Phys. Chem. Lett..

[cit49] Ptasinska S., Denifl S., Scheier P., Illenberger E., Märk T. D. (2005). Angew. Chem., Int. Ed..

[cit50] Fennimore M. A., Karsili T. N. V., Matsika S. (2017). Phys. Chem. Chem. Phys..

[cit51] Cooper C. D., Naff W. T., Compton R. N. (1975). J. Chem. Phys..

[cit52] Pshenichnyuk S. A., Asfandiarov N. L., Fal’ko V. S., Lukin V. G. (2003). Int. J. Mass..

[cit53] Chen J., Pelc A., Ameixa J., Kossoski F., Denifl S. (2024). ACS Omega.

[cit54] Ómarsson B., Ingólfsson O. (2013). Phys. Chem. Chem. Phys..

[cit55] Pshenichnyuk S. A., Vorob'ev A. S., Asfandiarov N. L., Modelli A. (2010). J. Chem. Phys..

[cit56] Khatymov R. V., Muftakhov M. V., Tuktarov R. F., Shchukin P. V., Khatymova L. Z., Pancras E., Terentyev A. G., Petrov N. I. (2024). J. Chem. Phys..

[cit57] Ptasińska S., Echt O., Denifl S., Stano M., Sulzer P., Zappa F., Stamatovic A., Scheier P., Märk T. D. (2006). J. Phys. Chem. A.

[cit58] Janečková R., Kubala D., May O., Fedor J., Allan M. (2013). Phys. Rev. Lett..

[cit59] Smith J. R., Kim J. B., Lineberger W. C. (1997). Phys. Rev. A.

[cit60] Modelli A., Pshenichnyuk S. A. (2013). Phys. Chem. Chem. Phys..

[cit61] Ameixa J., Arthur-Baidoo E., Pereira-da-Silva J., Ruivo J. C., Varella M. T. d. N., Beyer M. K., Ončák M., Ferreira da Silva F., Denifl S. (2022). ChemPhysChem.

[cit62] Mensa-Bonsu G., Wilson M. R., Tozer D. J., Verlet J. R. R. (2019). J. Chem. Phys..

[cit63] Ma Y. L., Li Q. M., Van den Heuvel H., Claeys M. (1997). Rapid Commun. Mass Spectrom..

[cit64] Nakata R., Yoshinaga N., Teraishi M., Okumoto Y., Huffaker A., Schmelz E. A., Mori N. (2018). Biosci. Biotechnol. Biochem..

[cit65] Spectral Database for Organic Compounds, SDBS, https://sdbs.db.aist.go.jp/, accessed on 29.7.2025

[cit66] Lengyel J., Rimarčík J., Vagánek A., Klein E. (2013). Phys. Chem. Chem. Phys..

[cit67] Ninh The S., Do Minh T., Nguyen Van T. (2019). J. Chem..

[cit68] Kobayashi T., Yokota K., Nagakura S. (1975). Bull. Chem. Soc. Jpn..

[cit69] NIST Chemistry WebBook, https://webbook.nist.gov/chemistry/, accessed on 08.10.2025

[cit70] Barbuch R. J., Coutant J. E., Welsh M. B., Setchell K. D. R. (1989). Biol. Mass Spectrom..

[cit71] Maul R., Schebb N. H., Kulling S. E. (2008). Anal. Bioanal. Chem..

[cit72] Turecek F., Hanus V. (1984). Mass Spectrom. Rev..

[cit73] Budzikiewicz H., Brauman J. I., Djerassi C. (1965). Tetrahedron.

[cit74] Kang J., Hick L. A., Price W. E. (2007). Rapid Commun. Mass Spectrom..

[cit75] Bowie J. H., Chai Ho A. (1975). J. Chem. Soc., Perkin Trans..

[cit76] Izadi F., Luxford T. F. M., Sedmidubská B., Arthur-Baidoo E., Kočišek J., Ončák M., Denifl S. (2024). Angew. Chem., Int. Ed..

