# Supplementary material for: Free electron interaction with genistein: positive and negative ion formation
Source: RSC Adv. 2025 Nov 26;15(54):46717–26. doi: 10.1039/d5ra05594f (PMC12649566; doi:10.1039/d5ra05594f)
Supplement: RA-015-D5RA05594F-s001 [file RA-015-D5RA05594F-s001.pdf]

Supplementary Information (SI) for RSC Advances.
This journal is © The Royal Society of Chemistry 2025

Supporting Information

for

Free Electron Interaction with Genistein: Positive and Negative Ion Formation

Vy T.T. Nguyen[1,2], Jiakuan Chen[1,2], Milan Ončák[1*], Stephan Denifl[1,2*]

[1]Institut für Ionenphysik und Angewandte Physik, Universität Innsbruck, Technikerstrasse 25, A-6020 Innsbruck, Austria.

[2]Center for Molecular Biosciences Innsbruck, Universität Innsbruck, Technikerstrasse 25, A-6020 Innsbruck, Austria.

*Corresponding authors: milan.oncak@uibk.ac.at, stephan.denifl@uibk.ac.at

Table S1 - List of cations from Genistein together with the assigned structure and calculated exponential factors. Uncertainties are related to the error resulting from the fitting.

Mass (u)	Assigned cations	Exponential factor *n**	
270	G^+	2.31 ± 0.05	–
269	$[G\text{-}H]^+$	1.85 ± 0.55	2.37 ± 0.15
153	$[^{1,3}A+H]^+$	1.58 ± 0.94	3.53 ± 0.43
152	$^{1,3}A^+$	2.06 ± 0.49	2.70 ± 0.50
118	$^{1,3}B^+$	2.46 ± 0.70	1.22 ± 0.23

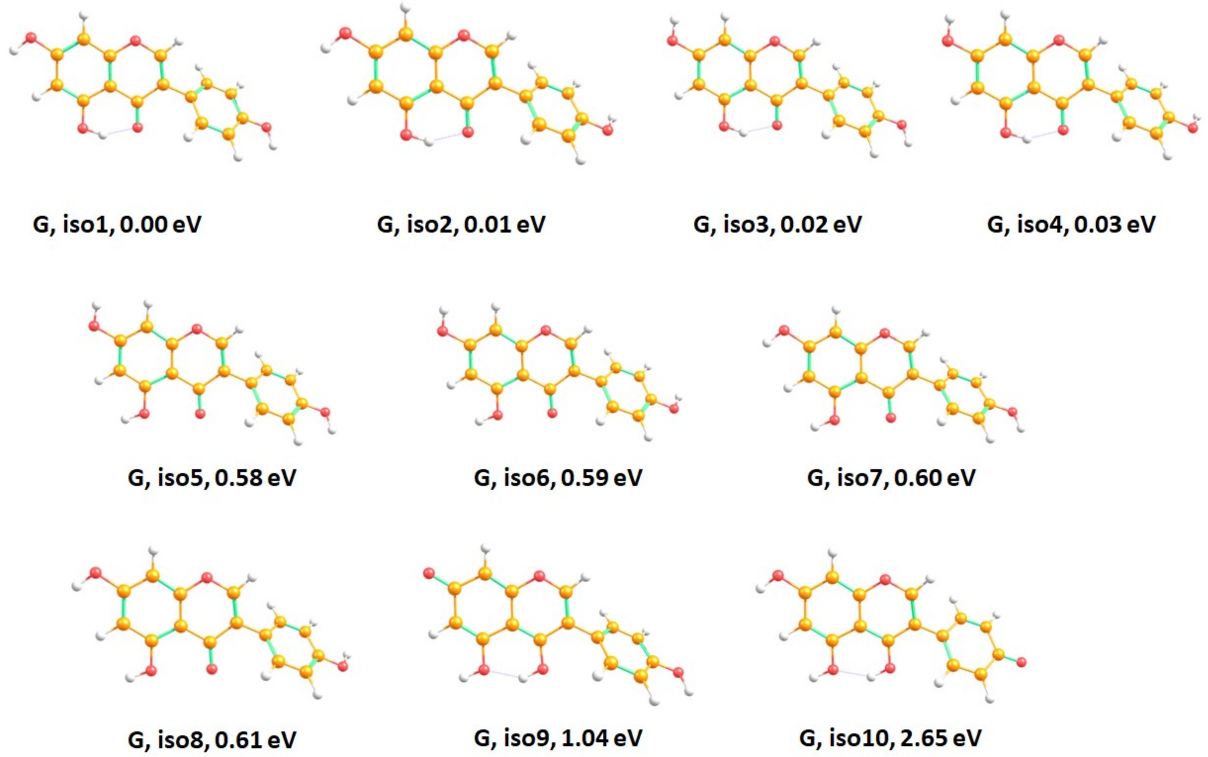

Figure S1 – Isomers of G as calculated at the ωB97XD/aug-cc-pVTZ//ωB97XD/aug-cc-pVDZ level of theory.

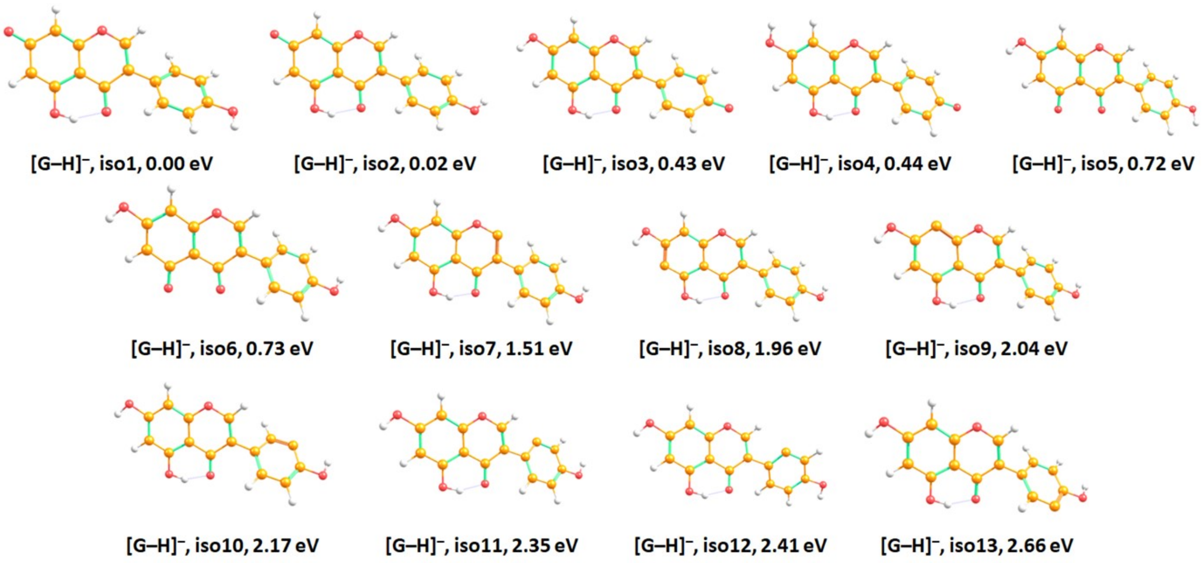

Figure S2 – Isomers of $[G–H]^-$ as calculated at the ωB97XD/aug-cc-pVTZ//ωB97XD/aug-cc-pVDZ level of theory.

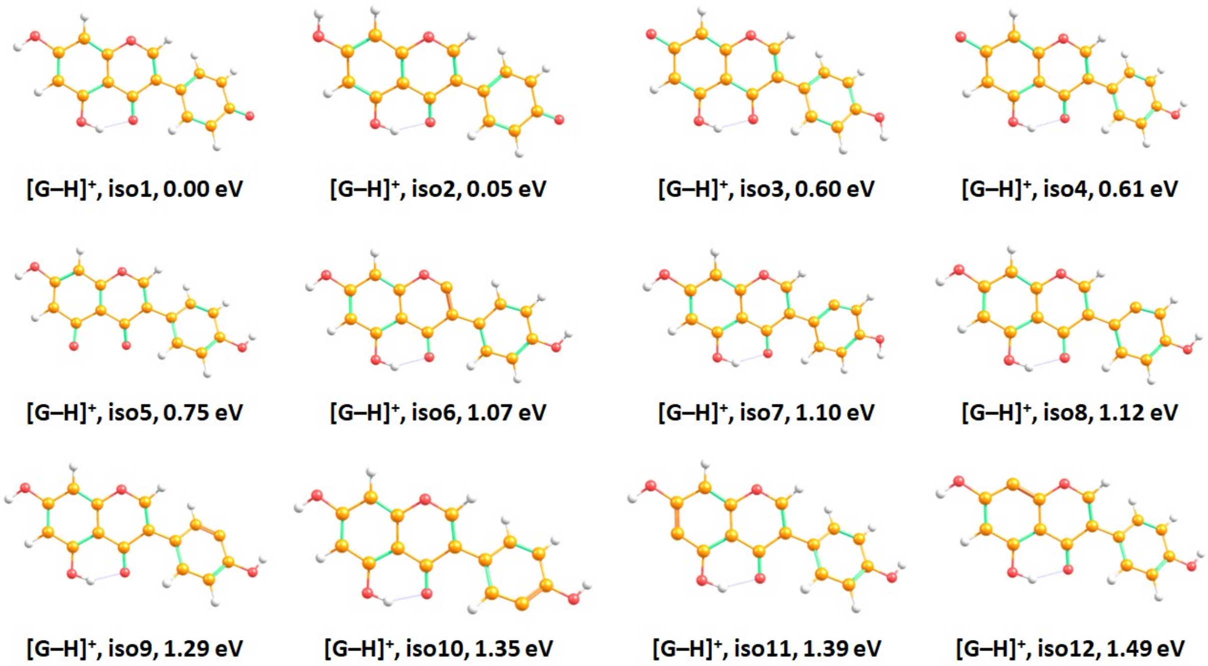

Figure S3 – Isomers of [G–H]$^+$ as calculated at the ωB97XD/aug-cc-pVTZ//ωB97XD/aug-cc-pVDZ level of theory.

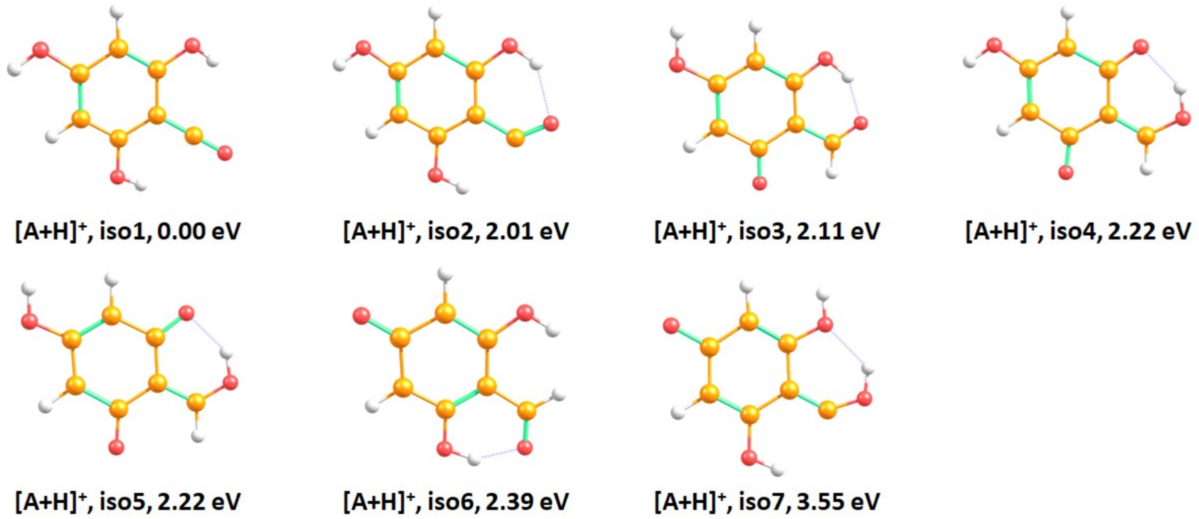

Figure S4 – Isomers of $[A+H]^+$ as calculated at the ωB97XD/aug-cc-pVTZ//ωB97XD/aug-cc-pVDZ level of theory.

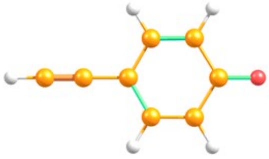

[B–H], iso1, 0.00 eV

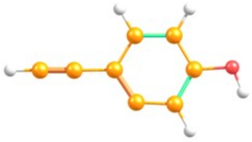

[B–H], iso2, 1.24 eV

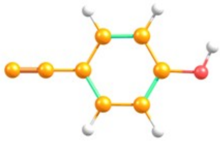

[B–H], iso3, 1.74 eV

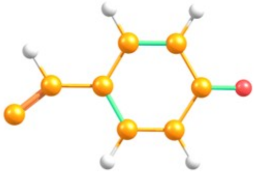

[B–H], iso4, 2.01 eV

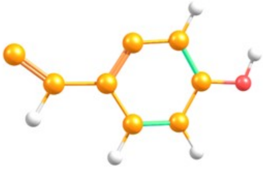

[B–H], iso5, 3.22 eV

Figure S5 – Isomers of [B–H] as calculated at the ωB97XD/aug-cc-pVTZ//ωB97XD/aug-cc-pVDZ level of theory.

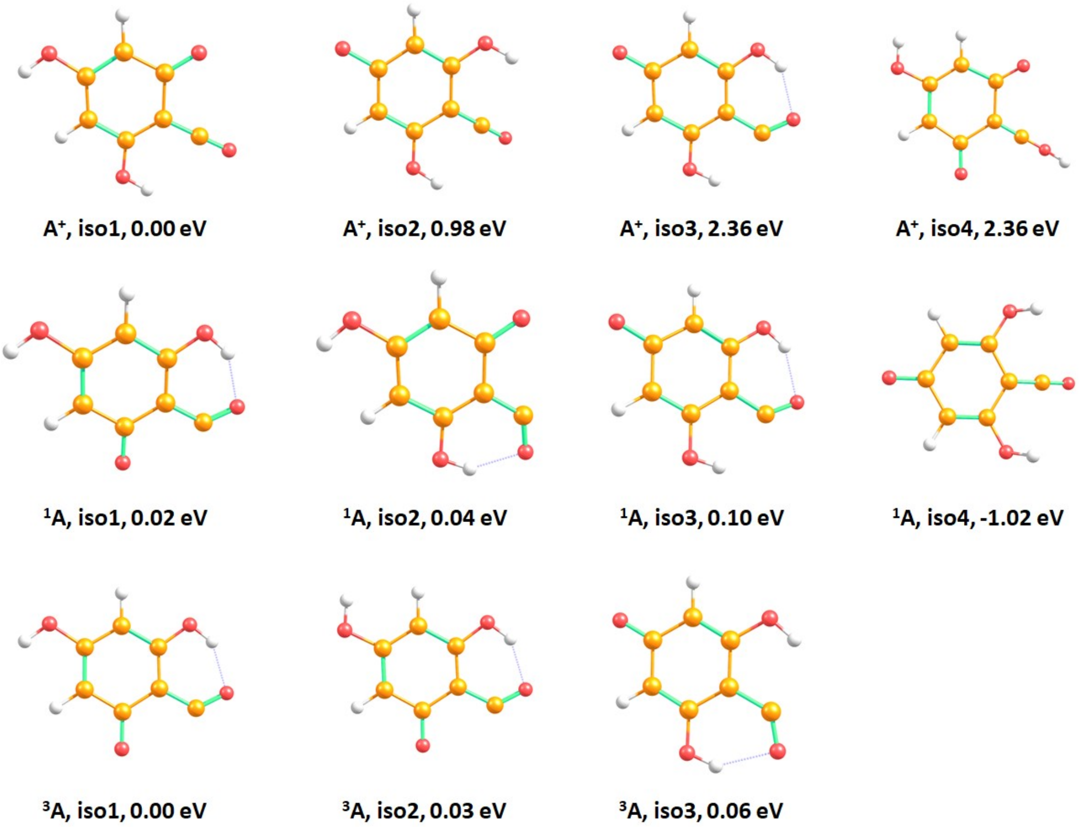

Figure S6 – Isomers of A^+ and A as calculated at the ωB97XD/aug-cc-pVTZ//ωB97XD/aug-cc-pVDZ level of theory. For A isomers, both singlet (^{1}A) and triplet (^{3}A) structures are shown.

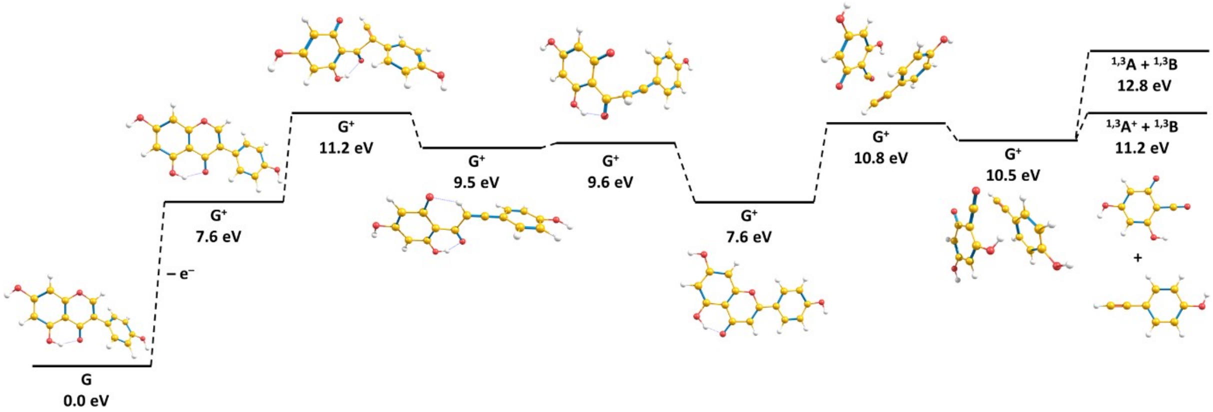

Figure S7 – Full reaction pathway to form $^{1,3}A^{+}$ + $^{1,3}B$ and $^{1,3}A$ + $^{1,3}B^{+}$ from G as calculated at the ωB97XD/aug-cc-pVTZ//ωB97XD/aug-cc-pVDZ level of theory.

Cartesian coordinates (in Å) and electronic energies including zero-point energy (in Hartree) as calculated at the ωB97XD/aug-cc-pVDZ level

G, iso1
E = -953.338549
C 2.909212 -1.134235 0.711396
C 2.106179 -0.265031 -0.038293
C 2.730917 0.771039 -0.742000
C 4.113305 0.919155 -0.715461
C 4.898018 0.032452 0.025626
C 4.292388 -0.998119 0.743914
C 0.637900 -0.453286 -0.092867
C 0.116919 -1.688296 -0.272234
O -1.191309 -1.999630 -0.296711
C -2.114823 -1.009771 -0.142496
C -1.704105 0.321423 0.026012
C -0.294414 0.672144 0.049294
C -2.713516 1.312528 0.176124
C -4.056302 0.952225 0.155415
C -4.407709 -0.391852 -0.015089
C -3.447749 -1.391995 -0.167173
O -2.385447 2.594496 0.338725
O -5.703424 -0.787229 -0.041660
O 0.084421 1.847509 0.186402
O 6.255645 0.130298 0.091246
H 2.443198 -1.923833 1.302047
H 4.911780 -1.672714 1.332778
H 4.582944 1.732081 -1.272602
H 2.130116 1.473548 -1.315653
H 0.728797 -2.575037 -0.423695
H 6.550641 0.883706 -0.426935
H -4.813169 1.727274 0.272532
H -3.734923 -2.431121 -0.300733
H -6.278798 -0.025542 0.071633
H -1.397001 2.637782 0.323599

G, iso2
E = -953.338234
C 3.445733 -1.395334 0.166234
C 2.113379 -1.011130 0.142656
C 1.704658 0.320585 -0.026653
C 2.715309 1.309974 -0.179060
C 4.057649 0.947605 -0.159431
C 4.407081 -0.396740 0.012168
O 1.188442 -1.999745 0.298409
C -0.119426 -1.686133 0.275255
C -0.638168 -0.450482 0.095715
C 0.295416 0.673598 -0.049116
C -2.106134 -0.258777 0.043046
C -2.731706 0.764565 0.770074
C -4.110776 0.918346 0.748353
C -4.896468 0.049792 -0.012916
C -4.292499 -0.965792 -0.753785
C -2.906895 -1.108831 -0.724912
O -6.244366 0.250031 0.001367
O -0.082418 1.848650 -0.188514
O 2.389066 2.592146 -0.342879
O 5.702221 -0.794258 0.037790
H -2.443080 -1.887288 -1.331805
H -4.898138 -1.637303 -1.365164
H -4.596001 1.711261 1.315338
H -2.128573 1.451558 1.359969
H -0.732931 -2.571529 0.428162
H -6.674275 -0.399446 -0.561131
H 4.815570 1.721390 -0.278139
H 3.731562 -2.434721 0.300663
H 6.278705 -0.033513 -0.076247
H 1.400692 2.637514 -0.326797

G, iso3
E = -953.337841
C -3.451800 -1.375616 -0.164421
C -2.115229 -0.998136 -0.140159
C -1.704598 0.330636 0.027526
C -2.715611 1.324375 0.177290
C -4.058066 0.969630 0.156948
C -4.411218 -0.371838 -0.012753
O -1.197332 -1.992563 -0.294218
C 0.112908 -1.685676 -0.270620
C 0.636056 -0.451972 -0.091990
C -0.293437 0.676942 0.050005
C 2.104765 -0.266923 -0.038187
C 2.731323 0.767116 -0.743291
C 4.114014 0.912219 -0.717512
C 4.897111 0.024591 0.024234
C 4.289614 -1.003892 0.743968
C 2.906137 -1.137034 0.712180
O 6.254883 0.119706 0.089435
O 0.090458 1.850062 0.186220
O -2.381760 2.604884 0.339192
O -5.737670 -0.648997 -0.023541
H 2.438587 -1.924799 1.304075
H 4.907845 -1.678999 1.333457
H 4.585142 1.723658 -1.275534
H 2.131762 1.470446 -1.317210
H 0.721715 -2.574486 -0.421943
H 6.551518 0.871133 -0.430705
H -4.826948 1.729467 0.271723
H -3.720982 -2.421812 -0.299048
H -5.874630 -1.592534 -0.145042
H -1.394440 2.646658 0.324219

G, iso4
E = -953.337513
C 3.449836 -1.378963 0.163551
C 2.113828 -0.999507 0.140281
C 1.705173 0.329756 -0.028206
C 2.717394 1.321800 -0.180267
C 4.059426 0.964996 -0.161034
C 4.410654 -0.376742 0.009878
O 1.194488 -1.992691 0.295753
C -0.115373 -1.683542 0.273416
C -0.636320 -0.449175 0.094627
C 0.294447 0.678390 -0.049860
C -2.104695 -0.260655 0.042872
C -2.732035 0.760686 0.771189
C -4.111440 0.911364 0.750419
C -4.895639 0.041916 -0.011388
C -4.289810 -0.971536 -0.753784
C -2.903900 -1.111618 -0.725682
O -6.243869 0.239211 0.003770
O -0.088462 1.851240 -0.188175
O 2.385262 2.602476 -0.343435
O 5.736713 -0.656058 0.019669
H -2.438584 -1.888163 -1.333889
H -4.894269 -1.643483 -1.365820
H -4.598081 1.702753 1.318306
H -2.130099 1.448673 1.361121
H -0.725852 -2.570981 0.426196
H -6.672667 -0.410004 -0.559868
H 4.829317 1.723567 -0.277382
H 3.717670 -2.425391 0.299103
H 5.872232 -1.599561 0.142971
H 1.398010 2.646144 -0.327461

G, iso5
E = -953.317094
C -3.419296 -1.394021 -0.166984
C -2.090481 -0.971559 -0.145199
C -1.700993 0.366098 0.023056
C -2.752518 1.310486 0.181589
C -4.083589 0.912865 0.165190
C -4.410418 -0.435196 -0.009408
O -1.176771 -1.965873 -0.306584
C 0.138435 -1.663479 -0.278087
C 0.653848 -0.432430 -0.099761
C -0.265978 0.725655 0.026831

C 2.123132 -0.252974 -0.038207
C 2.761335 0.773119 -0.745190
C 4.145023 0.908120 -0.712539
C 4.918158 0.020074 0.038808
C 4.300054 -0.999503 0.761175
C 2.915390 -1.123188 0.721635
O 6.277515 0.107774 0.110386
O 0.148639 1.869664 0.124741
O -2.424515 2.605967 0.352004
O -5.731245 -0.742672 -0.014869
H 2.438927 -1.904014 1.315661
H 4.909996 -1.675130 1.358759
H 4.624909 1.713492 -1.272161
H 2.168495 1.478512 -1.322762
H 0.743401 -2.555250 -0.426994
H 6.578754 0.856080 -0.411454
H -4.886809 1.639167 0.287108
H -3.644861 -2.450176 -0.304543
H -5.845280 -1.688784 -0.140272
H -3.229137 3.122824 0.453777

G, iso6
E = -953.316623
C 3.417565 -1.397152 0.166123
C 2.089202 -0.973392 0.142548
C 1.701458 0.364688 -0.026515
C 2.754135 1.308085 -0.183079
C 4.084838 0.909112 -0.164523
C 4.409973 -0.439270 0.010315
O 1.174303 -1.967081 0.302721
C -0.140676 -1.662715 0.276271
C -0.654066 -0.431071 0.098149
C 0.266664 0.725731 -0.033762
C -2.123008 -0.247433 0.041117
C -2.759301 0.767699 0.770971
C -4.139802 0.909537 0.746796
C -4.916559 0.039050 -0.021071
C -4.302663 -0.967053 -0.766023
C -2.915617 -1.099001 -0.733534
O -6.267423 0.227952 -0.009796
O -0.147935 1.868754 -0.139331
O 2.427460 2.603769 -0.353195
O 5.730445 -0.748208 0.017928
H -2.443644 -1.870047 -1.343663
H -4.901212 -1.639638 -1.383419
H -4.632723 1.696207 1.315975
H -2.161620 1.458320 1.361560
H -0.747007 -2.553316 0.426863
H -6.688168 -0.421395 -0.579170
H 4.889023 1.634669 -0.284651
H 3.641925 -2.453518 0.304204
H 5.843268 -1.694317 0.144289
H 3.232509 3.120653 -0.451297

G, iso7
E = -953.316259
C -3.414821 -1.408623 -0.169680
C -2.090099 -0.981690 -0.147868
C -1.700793 0.358666 0.021298
C -2.750663 1.300076 0.180767
C -4.082720 0.896635 0.164526
C -4.407531 -0.454280 -0.011168
O -1.171319 -1.971486 -0.309945
C 0.142136 -1.665387 -0.280387
C 0.655643 -0.433345 -0.101220
C -0.266464 0.721971 0.025041
C 2.124567 -0.251193 -0.038604
C 2.761692 0.776277 -0.744519
C 4.145155 0.913730 -0.710592
C 4.919238 0.026617 0.040828
C 4.302232 -0.994430 0.762078
C 2.917819 -1.120441 0.721409
O 6.278430 0.116678 0.113599
O 0.144327 1.867621 0.122834
O -2.427027 2.597205 0.352086
O -5.693552 -0.884674 -0.033430
H 2.442272 -1.902582 1.314441
H 4.912842 -1.669564 1.359563
H 4.624111 1.720175 -1.269518
H 2.168177 1.480805 -1.322480
H 0.749631 -2.555468 -0.429444
H 6.578868 0.864744 -0.409019
H -4.874411 1.638534 0.289498
H -3.657700 -2.458695 -0.306431
H -6.291918 -0.142354 0.085669
H -3.231091 3.114151 0.455138

G, iso8
E = -953.315816
C 3.413096 -1.411747 0.168648
C 2.088783 -0.983551 0.144892
C 1.701215 0.357187 -0.025012
C 2.752271 1.297698 -0.181900
C 4.083937 0.893005 -0.163180
C 4.407040 -0.458283 0.012380
O 1.168866 -1.972805 0.305543
C -0.144353 -1.664715 0.278317
C -0.655857 -0.432106 0.099254
C 0.267097 0.721872 -0.032869
C -2.124441 -0.245717 0.041380
C -2.759354 0.771483 0.769491
C -4.139609 0.915913 0.744334
C -4.917537 0.045741 -0.022642
C -4.305072 -0.962549 -0.765779
C -2.918283 -1.096917 -0.732481
O -6.268148 0.237067 -0.012364
O -0.143692 1.866443 -0.139329
O 2.429893 2.595076 -0.352585
O 5.692570 -0.890055 0.036867
H -2.447436 -1.869830 -1.341101
H -4.904522 -1.635115 -1.382326
H -4.631407 1.704164 1.312309
H -2.160801 1.461670 1.359721
H -0.753176 -2.553605 0.429214
H -6.689723 -0.413102 -0.580179
H 4.876629 1.634235 -0.285899
H 3.654623 -2.462083 0.305818
H 6.291935 -0.148164 -0.079926
H 3.234376 3.112130 -0.451687

G, iso9
E = -953.300341
C -3.459788 -1.440271 -0.173949
C -2.157904 -1.062617 -0.140807
C -1.716257 0.302976 0.028211
C -2.773737 1.279444 0.167435
C -4.081291 0.939613 0.139170
C -4.520482 -0.453060 -0.034866
C -0.362021 0.581708 0.054467
C 0.596802 -0.491720 -0.086710
C 0.092879 -1.736818 -0.251097
O -1.206766 -2.043465 -0.276721
C 2.064404 -0.276633 -0.038999
C 2.859638 -1.029165 0.833735
C 4.241728 -0.879788 0.864334
C 4.852588 0.044752 0.017584
C 4.074615 0.811934 -0.853616
C 2.694054 0.649116 -0.877648
O 6.207480 0.159020 0.087278
O 0.149035 1.799233 0.215791
O -2.342434 2.576169 0.329769
O -5.716939 -0.745726 -0.058519
H 2.387178 -1.736795 1.515640
H 4.856839 -1.464432 1.546384
H 4.548589 1.536752 -1.518058
H 2.100338 1.249578 -1.565160
H 0.716643 -2.617573 -0.385565
H -4.865236 1.691388 0.245267
H -3.732628 -2.484886 -0.304332
H -3.097321 3.163740 0.422974
H -0.579126 2.436618 0.312144
H 6.510353 0.826929 -0.533540

G, iso10
E = -953.241572
C 3.364165 -1.461539 0.120655
C 2.035509 -1.030984 0.121510
C 1.650850 0.324040 0.008853
C 2.721347 1.264011 -0.111397
C 4.034717 0.865133 -0.117191
C 4.356083 -0.510029 -0.000085
C 0.265021 0.647770 -0.015075
C -0.730923 -0.381964 0.081524
C -0.200854 -1.650898 0.270225
O 1.096007 -1.972454 0.246586
C -2.155758 -0.188605 -0.004604
C -2.978152 -1.241566 -0.505187
C -4.342821 -1.158155 -0.528090
C -5.055342 0.007777 -0.018958
C -4.191637 1.069321 0.480749
C -2.826277 0.974897 0.475417
O -6.296010 0.091790 -0.023171
O -0.158913 1.884765 -0.157315
O 2.363020 2.575093 -0.219651
O 5.636478 -0.936273 -0.000793
H -2.507006 -2.128393 -0.940395
H -4.944256 -1.961911 -0.954357
H -4.682607 1.961713 0.871056
H -2.247488 1.801079 0.885249
H -0.818086 -2.526954 0.442390
H 4.830531 1.606368 -0.208937
H 3.602745 -2.517467 0.213681
H 6.245534 -0.197851 -0.088564
H 3.130826 3.146135 -0.310836
H 0.589576 2.498665 -0.224617

G-
E = -953.363696
C 2.952140 -1.297390 0.403432
C 2.096181 -0.254813 -0.000840
C 2.710358 0.948253 -0.395710
C 4.096199 1.089287 -0.400425
C 4.916777 0.033147 -0.006021
C 4.339650 -1.165302 0.399911
C 0.630181 -0.433949 -0.028012
C 0.131055 -1.756001 -0.093877
O -1.231851 -2.042573 -0.185410
C -2.125656 -1.031825 -0.089965
C -1.704876 0.299382 0.023594
C -0.267629 0.649477 0.043526
C -2.697597 1.300238 0.112729
C -4.059798 0.963135 0.086835
C -4.425715 -0.375093 -0.028839
C -3.476122 -1.389538 -0.119156
O -2.341937 2.586253 0.221172
O -5.749828 -0.752154 -0.056909
O 0.045127 1.901472 0.143861
O 6.293880 0.131248 0.005315
H 2.520317 -2.233322 0.757074
H 4.980976 -1.985738 0.722885
H 4.540627 2.038578 -0.713628
H 2.076905 1.783123 -0.684466
H 0.711950 -2.604636 -0.443928
H 6.532064 1.014882 -0.284196
H -4.804197 1.757789 0.158383
H -3.772589 -2.431920 -0.207714
H -6.283722 0.042305 0.015943
H -1.316350 2.550916 0.212334

[G-H]-, iso1
E = -952.812169
C -3.479312 -1.406206 -0.165735
C -2.168801 -1.023995 -0.133503
C -1.735212 0.322426 0.031771
C -2.778342 1.308129 0.166806
C -4.102143 0.955660 0.138972
C -4.537948 -0.419919 -0.027471
C -0.357562 0.667966 0.063473
C 0.588230 -0.464702 -0.066973
C 0.079976 -1.703548 -0.243305
O -1.219008 -2.022306 -0.280481
C 2.055378 -0.271296 -0.020643
C 2.882542 -1.164800 0.673511
C 4.268849 -1.026043 0.685998
C 4.855887 0.039159 0.009281
C 4.052614 0.955819 -0.670422
C 2.669436 0.801662 -0.681482
O 6.224206 0.149576 0.050167
O 0.065097 1.847953 0.198174
O -2.421326 2.604043 0.325129
O -5.747346 -0.740295 -0.052575
H 2.430083 -1.981006 1.237873
H 4.900110 -1.725973 1.232442
H 4.508569 1.800374 -1.193108
H 2.049059 1.529376 -1.199179
H 0.703717 -2.584707 -0.390830
H 6.483416 0.937579 -0.433403
H -4.865832 1.725685 0.244805
H -3.749111 -2.452683 -0.298574
H -1.434552 2.617709 0.314990

[G-H]-, iso2
E = -952.811587
C -3.475218 -1.412118 -0.163761
C -2.165931 -1.025236 -0.136526
C -1.736601 0.322453 0.029162
C -2.782505 1.304156 0.170357
C -4.105238 0.946892 0.147889
C -4.536701 -0.429914 -0.018814
O -1.213251 -2.020285 -0.288950
C 0.084711 -1.697090 -0.253645
C 0.588752 -0.456772 -0.076529
C -0.359835 0.672929 0.057145
C 2.055279 -0.259304 -0.028674
C 2.673027 0.802596 -0.709040
C 4.053735 0.961576 -0.700378
C 4.854405 0.058765 -0.000541
C 4.265298 -0.993157 0.696391
C 2.877338 -1.137669 0.685004
O 6.212853 0.259381 -0.022217
O 0.060101 1.853342 0.192544
O -2.429403 2.600906 0.329567
O -5.745084 -0.754887 -0.038560
H 2.424848 -1.943744 1.263627
H 4.883093 -1.692409 1.265094
H 4.525340 1.787627 -1.231722
H 2.051862 1.518014 -1.242873
H 0.711707 -2.575678 -0.403170
H 6.634127 -0.423382 0.505200
H -4.871181 1.714024 0.258430
H -3.741856 -2.459342 -0.297270
H -1.442761 2.618584 0.315380

[G-H]-, iso3
E = -952.796462
C -3.398018 -1.423519 -0.143845
C -2.061329 -1.018765 -0.138174
C -1.675991 0.320753 0.003111
C -2.703662 1.295240 0.146826
C -4.041222 0.912529 0.142616
C -4.369190 -0.442047 -0.002427
O -1.130151 -1.981010 -0.282723
C 0.188745 -1.634676 -0.289016
C 0.706219 -0.390576 -0.092102
C -0.267613 0.704298 0.026647
C 2.155473 -0.196866 0.001127
C 2.816908 0.915241 -0.569652
C 4.192490 1.021755 -0.570228
C 5.059898 0.022254 0.019123
C 4.350120 -1.086673 0.623127
C 2.973550 -1.180097 0.603554
O 6.316105 0.114673 0.020325
O 0.050774 1.901490 0.156574
O -2.390776 2.584402 0.287718

O -5.671738 -0.851399 -0.011045
H 2.498814 -2.030167 1.104779
H 4.949312 -1.851149 1.122781
H 4.674897 1.889593 -1.025498
H 2.223363 1.704345 -1.032236
H 0.807820 -2.510924 -0.457901
H -4.811405 1.676316 0.252355
H -3.664711 -2.470705 -0.257444
H -6.238777 -0.083407 0.094479
H -1.391308 2.617752 0.267776

[G-H]-, iso4
E = -952.796171
C 2.968996 -1.183211 0.604121
C 2.153867 -0.196491 0.003170
C 2.818891 0.913629 -0.567567
C 4.194826 1.014523 -0.570108
C 5.059159 0.010891 0.016901
C 4.345865 -1.095354 0.621818
C 0.704326 -0.386237 -0.090332
C 0.185152 -1.629271 -0.287554
O -1.135608 -1.971893 -0.280645
C -2.061602 -1.005168 -0.136095
C -1.677103 0.332420 0.004521
C -0.267245 0.712168 0.027652
C -2.707146 1.308400 0.147647
C -4.045462 0.931706 0.143857
C -4.373459 -0.419736 -0.000180
C -3.401273 -1.404477 -0.141184
O -2.389251 2.596376 0.287633
O -5.703649 -0.727628 0.003359
O 0.055669 1.907494 0.156599
O 6.315653 0.097807 0.015668
H 2.491566 -2.031393 1.106018
H 4.942758 -1.862163 1.120652
H 4.680057 1.880804 -1.025299
H 2.227739 1.705421 -1.028541
H 0.801700 -2.507183 -0.456724
H -4.826053 1.680795 0.251596
H -3.651915 -2.458334 -0.255810
H -5.803789 -1.677222 -0.100665
H -1.391324 2.629280 0.267863

[G-H]-, iso5
E = -952.785461
C -3.423880 -1.382350 -0.169948
C -2.111490 -0.937360 -0.135080
C -1.715052 0.407450 0.036978
C -2.776842 1.428636 0.190947
C -4.135978 0.922376 0.152124
C -4.426271 -0.409859 -0.019660
C -0.298565 0.747644 0.056042
C 0.631589 -0.423174 -0.071324
C 0.121366 -1.653290 -0.257953
O -1.184983 -1.949920 -0.297426
C 2.100869 -0.248293 -0.019564
C 2.914106 -1.155522 0.673156
C 4.302857 -1.036618 0.691324
C 4.906123 0.022754 0.020631
C 4.117554 0.953001 -0.657761
C 2.732120 0.819886 -0.673219
O 6.277228 0.115673 0.065184
O 0.175147 1.878148 0.164872
O -2.553968 2.643034 0.346834
O -5.725096 -0.869151 -0.055379
H 2.448374 -1.967196 1.233530
H 4.922378 -1.746978 1.238035
H 4.587007 1.794220 -1.174270
H 2.120524 1.560118 -1.182898
H 0.730660 -2.543511 -0.413759
H 6.544196 0.907506 -0.407700
H -4.932249 1.662374 0.266814
H -3.659804 -2.433398 -0.310687
H -6.298754 -0.107103 0.058087

[G-H]-, iso6
E = -952.785018
C -3.419826 -1.387447 -0.170116
C -2.108726 -0.938286 -0.137470
C -1.716353 0.407501 0.036404
C -2.781102 1.424426 0.197540
C -4.138770 0.913998 0.159763
C -4.425079 -0.418652 -0.015181
O -1.179553 -1.948014 -0.303818
C 0.126056 -1.647256 -0.265419
C 0.631918 -0.415552 -0.079190
C -0.300725 0.752676 0.051096
C 2.100542 -0.235950 -0.026913
C 2.735069 0.816797 -0.707246
C 4.118264 0.954957 -0.695687
C 4.904314 0.042998 0.008703
C 4.299175 -0.998311 0.707112
C 2.908735 -1.123230 0.691301
O 6.267295 0.223522 -0.009410
O 0.171428 1.883497 0.159404
O -2.561647 2.638637 0.358830
O -5.722508 -0.882163 -0.049133
H 2.443024 -1.921087 1.271084
H 4.905692 -1.704165 1.280215
H 4.602831 1.773992 -1.226452
H 2.122987 1.541323 -1.238769
H 0.738553 -2.535257 -0.421808
H 6.673947 -0.460565 0.527524
H -4.937139 1.651073 0.278586
H -3.652618 -2.438970 -0.312610
H -6.298361 -0.122185 0.067062

[G-H]-, iso7
E = -952.758135
C -3.450940 -1.412829 -0.137057
C -2.100518 -1.037372 -0.101213
C -1.705208 0.294825 0.030854
C -2.709435 1.292927 0.135739
C -4.056057 0.938227 0.103002
C -4.404907 -0.413068 -0.033645
O -1.181406 -2.019845 -0.199237
C 0.191032 -1.807576 -0.182137
C 0.617572 -0.477099 -0.054752
C -0.285794 0.632043 0.056869
C 2.093405 -0.262668 -0.028570
C 2.707146 0.910369 -0.498582
C 4.093370 1.067996 -0.494890
C 4.906727 0.048654 -0.012109
C 4.325747 -1.125505 0.465259
C 2.940519 -1.271269 0.456185
O 6.273645 0.244145 -0.019820
O 0.045211 1.850685 0.182271
O -2.360788 2.577786 0.266156
O -5.724165 -0.792398 -0.068604
H 2.485828 -2.194594 0.812332
H 4.956243 -1.932327 0.850535
H 4.551235 1.985359 -0.865807
H 2.085166 1.721629 -0.867526
H 6.688978 -0.544667 0.335963
H -4.815131 1.717562 0.184596
H -3.732775 -2.457557 -0.241451
H -6.265734 -0.003877 0.012711
H -1.349729 2.559551 0.262802

[G-H]-, iso8
E = -952.741158
C -3.465488 -1.382899 -0.165264
C -2.142648 -1.001293 -0.136804
C -1.747176 0.345885 0.034177
C -2.806294 1.318691 0.177960
C -4.161726 1.010104 0.160863
C -4.413403 -0.351100 -0.012583
O -1.203897 -2.004830 -0.293002
C 0.090364 -1.689735 -0.258653
C 0.594460 -0.448269 -0.078417
C -0.346320 0.684793 0.058800
C 2.062331 -0.255517 -0.030449

C 2.686135 0.796106 -0.720848
C 4.067478 0.948851 -0.711059
C 4.862485 0.050080 0.000448
C 4.267239 -0.991886 0.707047
C 2.878676 -1.130396 0.693618
O 6.221621 0.244402 -0.020420
O 0.084929 1.850789 0.193090
O -2.429466 2.617159 0.341737
O -5.727893 -0.754920 -0.042570
H 2.421260 -1.928886 1.278960
H 4.880631 -1.687886 1.284399
H 4.543882 1.767039 -1.250156
H 2.070063 1.508566 -1.264495
H 0.716889 -2.568425 -0.410151
H 6.638798 -0.433153 0.516887
H -3.746402 -2.426263 -0.301266
H -6.192688 0.087610 0.075527
H -1.451458 2.640110 0.326612

[G-H]-, iso9
E = -952.738331
C -3.481479 -1.525974 -0.178854
C -2.190785 -1.050880 -0.143731
C -1.736427 0.292782 0.022295
C -2.746725 1.278943 0.166025
C -4.074844 0.889857 0.141855
C -4.390779 -0.478419 -0.023323
O -1.182001 -2.031596 -0.303119
C 0.091719 -1.697084 -0.264656
C 0.604550 -0.450092 -0.082491
C -0.341279 0.659838 0.049277
C 2.070946 -0.254037 -0.031707
C 2.692878 0.820966 -0.687895
C 4.073991 0.979536 -0.673142
C 4.873094 0.060975 0.006987
C 4.281419 -1.005803 0.678554
C 2.893374 -1.148416 0.662872
O 6.232837 0.260167 -0.007981
O 0.048337 1.846888 0.184456
O -2.428488 2.585963 0.327791
O -5.737865 -0.809510 -0.031984
H 2.440828 -1.967876 1.222378
H 4.897636 -1.719281 1.231273
H 4.546704 1.816363 -1.186549
H 2.074691 1.547517 -1.210136
H 0.737607 -2.564134 -0.416147
H 6.651378 -0.437817 0.501311
H -4.842803 1.660790 0.254186
H -6.244288 -0.001124 0.084973
H -1.442225 2.617125 0.313008

[G-H]-, iso10
E = -952.733605
C 3.391276 -1.430175 0.154030
C 2.058295 -1.017405 0.134292
C 1.682139 0.322829 -0.016867
C 2.715142 1.290966 -0.155359
C 4.050843 0.900649 -0.138478
C 4.369746 -0.454468 0.015696
O 1.118902 -1.976749 0.274524
C -0.194664 -1.624052 0.260861
C -0.697318 -0.376064 0.083141
C 0.274629 0.714096 -0.043695
C -2.160649 -0.188966 0.009564
C -2.794315 0.872529 0.662830
C -4.186808 0.956348 0.638234
C -4.912769 -0.028636 -0.037634
C -4.359980 -1.109654 -0.746312
C -2.961696 -1.130845 -0.688835
O -6.291524 0.082028 -0.009629
O -0.038043 1.912386 -0.175637
O 2.409539 2.581040 -0.303225
O 5.669183 -0.872145 0.036763
H -2.419135 -1.911084 -1.243665
H -4.701239 1.776385 1.144982
H -2.211375 1.625839 1.191548
H -0.827744 -2.494734 0.407152
H -6.557653 -0.683356 -0.540416
H 4.826286 1.659421 -0.246007
H 3.650854 -2.478449 0.273612
H 6.242409 -0.108900 -0.069928
H 1.409909 2.619480 -0.288462

[G-H]-, iso11
E = -952.727264
C 3.410943 -1.439767 0.102755
C 2.076434 -1.025531 0.091076
C 1.698451 0.317178 -0.012377
C 2.732460 1.289591 -0.111756
C 4.068620 0.899122 -0.102431
C 4.388771 -0.460620 0.004731
O 1.135277 -1.986778 0.189364
C -0.174996 -1.634677 0.173772
C -0.679100 -0.377369 0.061744
C 0.288083 0.708983 -0.022687
C -2.167470 -0.240128 0.005183
C -2.769882 0.961248 0.413806
C -4.158849 1.095593 0.406174
C -4.920394 0.010131 -0.016460
C -4.300507 -1.180454 -0.411362
C -2.908314 -1.378663 -0.430025
O -6.298121 0.154731 -0.033804
O -0.007309 1.922808 -0.113869
O 2.425997 2.584501 -0.214091
O 5.691827 -0.875838 0.016768
H -4.970355 -1.998271 -0.730246
H -4.644900 2.021551 0.715073
H -2.163623 1.806906 0.737362
H -0.844404 -2.491814 0.214008
H -6.658067 -0.678519 -0.349588
H 4.843998 1.662069 -0.178878
H 3.668469 -2.492294 0.183988
H 6.260312 -0.105732 -0.060304
H 1.421701 2.612889 -0.199214

[G-H]-, iso12
E = -952.724936
C -3.416191 -1.432514 0.099938
C -2.080209 -1.022970 0.089529
C -1.697364 0.318615 -0.010417
C -2.727978 1.294965 -0.106872
C -4.065462 0.909239 -0.098749
C -4.390513 -0.449702 0.004483
O -1.142460 -1.987656 0.185921
C 0.168920 -1.640853 0.169988
C 0.677384 -0.384757 0.060094
C -0.285607 0.705017 -0.019511
C 2.166705 -0.254503 0.002894
C 2.903707 -1.402088 -0.417080
C 4.300891 -1.220774 -0.406429
C 4.922397 -0.027575 -0.030739
C 4.162695 1.068670 0.377389
C 2.772255 0.949947 0.392650
O 6.307253 0.042471 -0.075071
O 0.013804 1.918834 -0.105626
O -2.416831 2.589191 -0.205268
O -5.695073 -0.860232 0.015216
H 2.173252 1.804775 0.705242
H 4.644083 2.005514 0.675061
H 4.967512 -2.037444 -0.710927
H 0.837041 -2.499437 0.207568
H 6.563435 0.925142 0.202573
H -4.838146 1.675134 -0.173101
H -3.677367 -2.484354 0.178329
H -6.260772 -0.087845 -0.059414
H -1.412357 2.613351 -0.189720

[G-H]-, iso13
E = -952.715846
C -3.395290 -1.423803 -0.168029
C -2.061878 -1.014913 -0.134234
C -1.684149 0.323456 0.027479

C -2.714772 1.293217 0.165385
C -4.052050 0.906859 0.132878
C -4.372791 -0.445649 -0.033603
O -1.123353 -1.979145 -0.266827
C 0.191038 -1.625647 -0.245883
C 0.692764 -0.380463 -0.070056
C -0.274963 0.713306 0.069109
C 2.159102 -0.183812 -0.021433
C 2.815579 0.746657 -0.862187
C 4.208001 0.925195 -0.948662
C 4.906530 0.078703 -0.069939
C 4.319906 -0.850593 0.801063
C 2.930618 -0.987574 0.821252
O 6.300369 0.179390 -0.065423
O 0.042142 1.903178 0.231802
O -2.406584 2.580170 0.327506
O -5.672882 -0.860540 -0.069989
H 2.456786 -1.695295 1.506117
H 4.927599 -1.468269 1.474579
H 2.165056 1.357168 -1.501982
H 0.825073 -2.496752 -0.389396
H 6.637550 -0.449051 0.578610
H -4.826252 1.667206 0.238041
H -3.657238 -2.470423 -0.296854
H -6.245472 -0.095912 0.030215
H -1.406499 2.617280 0.325290

H
E = -0.502268974039
H 0.000000 0.000000 0.000000

[G-H]+, iso1
E = -952.423786
C -3.329446 -1.438633 -0.112231
C -2.035098 -1.000269 -0.058232
C -1.647229 0.347528 0.036066
C -2.694109 1.312479 0.082483
C -4.028357 0.902575 0.029408
C -4.339777 -0.451698 -0.066869
O -1.053872 -1.988200 -0.103446
C 0.200656 -1.688521 -0.041536
C 0.726888 -0.396217 0.041976
C -0.259416 0.734819 0.083913
C 2.137957 -0.239210 0.033964
C 2.733928 1.053612 -0.260966
C 4.073731 1.199777 -0.325236
C 4.990193 0.062731 -0.060434
C 4.358192 -1.237414 0.284795
C 3.019195 -1.368746 0.315148
O 6.194726 0.184108 -0.120662
O 0.091794 1.913140 0.183271
O -2.439133 2.608013 0.175748
O -5.594530 -0.909905 -0.121172
H 2.600315 -2.326548 0.613189
H 5.029130 -2.063357 0.520412
H 4.537741 2.152732 -0.579894
H 2.072188 1.892354 -0.446803
H 0.815632 -2.583189 -0.115303
H -4.806864 1.663361 0.067542
H -3.581556 -2.493014 -0.186053
H -6.234610 -0.190634 -0.086058
H -1.467693 2.734163 0.209568

[G-H]+, iso2
E = -952.421866
C 3.016956 -1.369178 0.324531
C 2.137570 -0.240965 0.034193
C 2.733731 1.048525 -0.272713
C 4.073798 1.192916 -0.338769
C 4.989122 0.057278 -0.063435
C 4.356192 -1.239458 0.293405
C 0.725602 -0.396844 0.044704
C 0.198251 -1.687267 -0.039814
O -1.058377 -1.982941 -0.102847
C -2.035060 -0.991485 -0.057787
C -1.647688 0.353229 0.038889
C -0.257884 0.736550 0.089017
C -2.695844 1.321002 0.085060
C -4.027556 0.913701 0.029012
C -4.342490 -0.439331 -0.069824
C -3.333687 -1.428185 -0.114602
O -2.436539 2.615920 0.180822
O -5.642070 -0.753065 -0.118431
O 0.097951 1.912380 0.192612
O 6.193704 0.176862 -0.125027
H 2.596118 -2.323348 0.631579
H 5.026553 -2.063627 0.536792
H 4.538743 2.142853 -0.602878
H 2.072631 1.886102 -0.466087
H 0.810626 -2.583599 -0.114953
H -4.820646 1.656787 0.065308
H -3.561744 -2.489484 -0.190119
H -5.779432 -1.703500 -0.185515
H -1.466275 2.741631 0.217644

[G-H]+, iso3
E = -952.402376
C 2.920813 -1.331122 0.384379
C 2.046914 -0.261322 -0.005277
C 2.648773 0.984755 -0.385026
C 4.008770 1.139933 -0.398614
C 4.842660 0.062645 -0.008269
C 4.276985 -1.179296 0.391815
C 0.619118 -0.438620 -0.022361
C 0.093424 -1.716572 -0.134033
O -1.173164 -2.023230 -0.177730
C -2.148576 -1.036311 -0.090685
C -1.755701 0.327343 0.020800
C -0.347126 0.691087 0.047677
C -2.773670 1.321940 0.111633
C -4.111789 0.939852 0.085023
C -4.489431 -0.447496 -0.031585
C -3.438351 -1.456213 -0.121082
O -2.485394 2.614042 0.221408
O -5.686822 -0.788950 -0.056274
O 0.019480 1.863806 0.150039
O 6.157793 0.140200 0.013341
H 2.510546 -2.276079 0.734705
H 4.943738 -1.978318 0.710415
H 4.452068 2.085868 -0.710933
H 2.010636 1.811043 -0.679931
H 0.711127 -2.606895 -0.239239
H 6.482275 1.008022 -0.262487
H -4.897794 1.689637 0.153030
H -3.711420 -2.505322 -0.208527
H -1.513204 2.721485 0.230334

[G-H]+, iso4
E = -952.401760
C -3.435834 -1.460701 -0.121573
C -2.147084 -1.038116 -0.090676
C -1.756905 0.326298 0.021882
C -2.776612 1.318702 0.113709
C -4.114210 0.933807 0.086493
C -4.489028 -0.454184 -0.031335
O -1.169378 -2.023033 -0.178060
C 0.096681 -1.713443 -0.135112
C 0.619802 -0.434698 -0.023435
C -0.349002 0.693247 0.049004
C 2.047036 -0.251577 -0.010358
C 2.647207 0.992398 -0.397145
C 4.005578 1.148067 -0.410714
C 4.842638 0.074860 -0.012264
C 4.279848 -1.164806 0.395754
C 2.922103 -1.318239 0.386369
O 6.141478 0.295674 -0.039236
O 0.015438 1.865809 0.154713
O -2.491341 2.611094 0.224993
O -5.685757 -0.797961 -0.056137
H 2.512425 -2.261619 0.741979
H 4.928443 -1.976586 0.725742
H 4.469282 2.081305 -0.724935

H 2.009027 1.816280 -0.697836
H 0.715827 -2.602596 -0.241969
H 6.655999 -0.471965 0.243629
H -4.901685 1.682005 0.155103
H -3.706781 -2.510262 -0.210051
H -1.519375 2.721101 0.234558

[G-H]+, iso5
E = -952.396492
C -3.414532 -1.355421 -0.118270
C -2.081549 -0.919789 -0.042611
C -1.690954 0.397500 0.068978
C -2.765583 1.411606 0.064704
C -4.140092 0.941803 0.003522
C -4.448800 -0.406675 -0.087526
O -1.165439 -1.950948 -0.085205
C 0.121193 -1.687503 -0.058740
C 0.666119 -0.424258 0.051506
C -0.269642 0.743702 0.210375
C 2.093492 -0.256485 0.008762
C 2.678151 0.999158 -0.368087
C 4.034805 1.157189 -0.425403
C 4.884128 0.074075 -0.085468
C 4.337532 -1.180796 0.300887
C 2.980955 -1.337078 0.333323
O 6.181739 0.296070 -0.148210
O 0.148793 1.852049 0.475431
O -2.530956 2.623033 0.084308
O -5.698343 -0.895725 -0.156861
H 2.583034 -2.292964 0.668009
H 4.999309 -2.000887 0.580375
H 4.487233 2.099076 -0.729909
H 2.026531 1.829501 -0.620126
H 0.705283 -2.599136 -0.168637
H 6.703628 -0.480470 0.093433
H -4.910766 1.712989 0.013472
H -3.639974 -2.417183 -0.196215
H -6.351971 -0.188921 -0.131808

[G-H]+, iso6
E = -952.384186
C -3.380295 -1.475513 0.000035
C -2.094038 -1.015485 0.000117
C -1.709663 0.334330 0.000057
C -2.766695 1.289280 -0.000099
C -4.097516 0.864787 -0.000161
C -4.398085 -0.495144 -0.000115
O -1.072316 -1.994793 0.000308
C 0.136588 -1.621692 0.000236
C 0.693458 -0.361968 0.000152
C -0.321181 0.747258 0.000115
C 2.118709 -0.209247 0.000126
C 2.758845 1.070652 0.000260
C 4.123595 1.169057 0.000169
C 4.919173 -0.005614 -0.000107
C 4.311810 -1.289463 -0.000249
C 2.949233 -1.380711 -0.000166
O 6.228241 0.166795 -0.000259
O 0.018161 1.931615 0.000205
O -2.523513 2.591377 -0.000177
O -5.650795 -0.965727 -0.000207
H 2.480135 -2.364637 -0.000362
H 4.930322 -2.187184 -0.000451
H 4.624675 2.135324 0.000290
H 2.143701 1.964840 0.000374
H 6.710402 -0.670296 -0.000471
H -4.882629 1.619631 -0.000271
H -3.620745 -2.535061 0.000119
H -6.294632 -0.249204 -0.000263
H -1.553082 2.729284 -0.000144

[G-H]+, iso7
E = -952.383681
C -3.365803 -1.453932 0.000050
C -2.067655 -1.013882 0.000109
C -1.692127 0.341988 0.000147
C -2.744420 1.303806 0.000108
C -4.077377 0.888691 0.000014
C -4.380069 -0.471120 -0.000032
O -1.090825 -1.994890 0.000158
C 0.171889 -1.672379 -0.000077
C 0.674573 -0.376291 -0.000142
C -0.308550 0.742532 0.000276
C 2.095360 -0.213089 -0.000492
C 2.968085 -1.327957 -0.000385
C 4.314145 -1.326287 0.000202
C 4.929185 -0.035484 0.000128
C 4.135214 1.140170 -0.000311
C 2.765826 1.059462 -0.000684
O 6.247438 -0.016715 0.000475
O 0.041751 1.929422 0.000489
O -2.486606 2.602160 -0.000012
O -5.634330 -0.937582 -0.000156
H 2.156777 1.959762 -0.000987
H 4.622440 2.115364 -0.000402
H 4.931995 -2.223714 0.000549
H 0.819443 -2.549812 -0.000216
H 6.602410 0.882234 0.000053
H -4.859732 1.646352 -0.000037
H -3.615007 -2.511654 0.000145
H -6.275511 -0.218703 -0.000151
H -1.511881 2.723635 -0.000070

[G-H]+, iso8
E = -952.383023
C 3.363130 -1.458886 -0.000027
C 2.066026 -1.015553 0.000122
C 1.693810 0.341047 0.000146
C 2.748286 1.300352 0.000003
C 4.080352 0.881906 -0.000173
C 4.379792 -0.478525 -0.000196
O 1.086617 -1.994118 0.000258
C -0.175396 -1.668180 0.000112
C -0.675191 -0.371223 0.000122
C 0.311098 0.745395 0.000364
C -2.095670 -0.202438 -0.000039
C -2.766190 1.070745 -0.000258
C -4.133783 1.152616 -0.000293
C -4.930276 -0.021597 -0.000111
C -4.317194 -1.313737 0.000066
C -2.969514 -1.315357 0.000039
O -6.238462 0.145645 -0.000207
O -0.035971 1.932697 0.000559
O 2.493805 2.599189 -0.000005
O 5.633031 -0.948061 -0.000364
H -4.914966 -2.226538 0.000140
H -4.640097 2.115832 -0.000437
H -2.156719 1.970493 -0.000342
H -0.824607 -2.544346 0.000070
H -6.721067 -0.691130 0.000003
H 4.864487 1.637736 -0.000289
H 3.609837 -2.517184 0.000012
H 6.275855 -0.230656 -0.000450
H 1.519433 2.723587 0.000110

[G-H]+, iso9
E = -952.376525
C 3.382600 -1.410121 0.103145
C 2.073206 -1.005491 0.071008
C 1.660438 0.334887 -0.023141
C 2.685558 1.322950 -0.092925
C 4.029682 0.944931 -0.062864
C 4.369869 -0.402354 0.034227
O 1.122174 -2.007919 0.138732
C -0.150448 -1.724365 0.102025
C -0.694513 -0.448981 0.008572
C 0.266170 0.695649 -0.052804
C -2.119986 -0.289248 0.001186
C -2.743820 0.952789 0.349307
C -4.107134 1.103149 0.357495
C -4.951367 0.019612 -0.000214
C -4.316653 -1.178580 -0.361938

C -2.985116 -1.387439 -0.370515
O -6.257851 0.200516 0.031026
O -0.113718 1.867750 -0.147790
O 2.393468 2.610066 -0.187158
O 5.636210 -0.833255 0.067889
H -2.585132 -2.339505 -0.717254
H -4.572857 2.043642 0.650725
H -2.112923 1.790534 0.626566
H -0.751747 -2.626738 0.194138
H -6.735484 -0.599488 -0.226144
H 4.790862 1.721912 -0.118388
H 3.660204 -2.458027 0.177769
H 6.257990 -0.099193 0.017783
H 1.415915 2.705257 -0.202152

[G-H]+, iso10
E = -952.374251
C -3.363595 -1.430491 -0.103288
C -2.060522 -1.005749 -0.074488
C -1.668896 0.340962 0.021716
C -2.708763 1.312914 0.096589
C -4.046953 0.914052 0.070016
C -4.366231 -0.438209 -0.028875
O -1.094050 -1.993107 -0.149679
C 0.174236 -1.690195 -0.114980
C 0.696788 -0.406583 -0.012444
C -0.280554 0.722137 0.049697
C 2.118256 -0.220050 0.001144
C 2.707451 1.039863 -0.385553
C 4.053109 1.125229 -0.410166
C 4.948233 0.110888 -0.044773
C 4.373388 -1.131022 0.347372
C 3.011060 -1.281993 0.364063
O 6.244978 0.352084 -0.066066
O 0.084175 1.899397 0.144723
O -2.436219 2.604101 0.192586
O -5.625724 -0.888876 -0.059449
H 2.613110 -2.229364 0.721522
H 5.022035 -1.948575 0.666008
H 2.069146 1.870031 -0.678564
H 0.790865 -2.581212 -0.215831
H 6.762170 -0.417204 0.205678
H -4.820036 1.678874 0.129662
H -3.625349 -2.482329 -0.179953
H -6.258845 -0.164799 -0.005980
H -1.460420 2.715276 0.204350

[G-H]+, iso11
E = -952.372841
C -3.451867 -1.394001 -0.116623
C -2.143103 -0.976176 -0.079984
C -1.729636 0.365005 0.029925
C -2.747811 1.367409 0.114101
C -4.052784 0.918100 0.074056
C -4.451788 -0.398209 -0.035817
O -1.189419 -1.976398 -0.161323
C 0.084340 -1.691519 -0.122227
C 0.626280 -0.419555 -0.014883
C -0.331379 0.722890 0.061695
C 2.054257 -0.255615 -0.007789
C 2.666018 0.985293 -0.384774
C 4.026427 1.126643 -0.403216
C 4.853106 0.041230 -0.021052
C 4.279572 -1.195854 0.375832
C 2.919707 -1.335319 0.372145
O 6.156212 0.247596 -0.052196
O 0.048785 1.893139 0.173187
O -2.463030 2.654368 0.223685
O -5.725037 -0.802767 -0.070534
H 2.502161 -2.278449 0.718958
H 4.921174 -2.018218 0.693081
H 4.498149 2.058395 -0.709651
H 2.032757 1.818041 -0.671754
H 0.685969 -2.592090 -0.228619
H 6.660592 -0.530319 0.219308
H -3.719385 -2.444536 -0.203156
H -6.323640 -0.049308 -0.009151
H -1.485906 2.747045 0.240100

[G-H]+, iso12
E = -952.369152
C -3.413456 -1.403526 -0.121166
C -2.103421 -1.048585 -0.087715
C -1.722210 0.305154 0.026696
C -2.764865 1.272273 0.109331
C -4.105723 0.880021 0.072316
C -4.449870 -0.471433 -0.044483
O -1.144071 -2.039794 -0.169758
C 0.125003 -1.727842 -0.126133
C 0.643755 -0.446903 -0.014630
C -0.330477 0.683447 0.063459
C 2.069358 -0.260642 -0.007552
C 2.661052 0.989342 -0.387908
C 4.018753 1.152618 -0.406466
C 4.862896 0.081340 -0.021029
C 4.309355 -1.164103 0.379284
C 2.952166 -1.325518 0.375594
O 6.162060 0.308614 -0.052685
O 0.033777 1.858022 0.180297
O -2.495254 2.566408 0.223399
O -5.707565 -0.919217 -0.085469
H 2.549343 -2.274017 0.725164
H 4.964198 -1.974944 0.699151
H 4.475721 2.090804 -0.715653
H 2.014211 1.810864 -0.677272
H 0.743292 -2.616975 -0.233229
H 6.679348 -0.460214 0.220743
H -4.874633 1.649895 0.138801
H -6.335588 -0.190902 -0.025152
H -1.521357 2.679385 0.243598

[A+H]+, iso1
E = -570.141624
C -1.941047 -0.018726 -0.050124
C -1.256732 -1.250815 0.000364
C 0.109458 -1.262317 0.075978
C 0.826278 0.004448 0.076928
C 0.092171 1.257812 0.076074
C -1.276397 1.225335 0.002174
O 0.730188 -2.441506 0.099793
C 2.170788 0.014446 -0.087154
O 3.301675 0.022480 -0.228570
O 0.688180 2.448883 0.103686
O -3.255866 -0.103131 -0.122247
H -1.825040 2.165719 -0.000203
H -1.813042 -2.184927 -0.005983
H -3.683012 0.762414 -0.145912
H 1.614019 2.442473 0.369573
H 1.646550 -2.420585 0.395783

[A+H]+, iso2
E = -570.077861
C 1.498491 1.056518 0.000026
C 1.965986 -0.247093 -0.000254
C 1.047265 -1.324917 -0.000008
C -0.371632 -1.066807 0.000047
C -0.858097 0.248148 -0.000584
C 0.087208 1.299567 -0.000002
O 3.248312 -0.596651 -0.000373
O -1.133569 -2.122774 0.001026
C -2.286739 0.527691 -0.000719
O -3.171226 -0.267685 -0.000669
O -0.266675 2.554820 0.000739
H 2.164646 1.918574 0.000537
H 1.400867 -2.355955 0.000146
H 3.843502 0.162847 -0.000657
H -1.235964 2.654290 0.001612
H -2.082680 -1.880088 0.001535

[A+H]+, iso3
E = -570.068824
6 1.567316 1.091714 0.000256

6 0.121194 1.415954 -0.000140
6 -0.850252 0.296137 0.000279
6 -0.361486 -1.037262 -0.000400
6 1.032763 -1.286592 -0.000192
6 1.986739 -0.248674 0.000225
8 -0.254079 2.570583 -0.001159
6 -2.300457 0.559609 0.001024
8 -3.087797 -0.375696 0.000525
8 -1.148071 -2.076894 -0.000917
8 3.288811 -0.464612 0.000561
1 1.348855 -2.332088 -0.000271
1 2.284478 1.912575 0.000255
1 3.527115 -1.401297 0.000430
1 -2.090085 -1.760478 -0.000529
1 -2.636177 1.608927 0.001713

[A+H]+, iso4
E = -570.064701
C 2.021297 -0.275770 -0.000001
C 1.544685 1.037591 0.000002
C 0.084999 1.386903 0.000064
C -0.874524 0.294986 0.000016
C -0.405080 -1.061836 0.000011
C 1.067228 -1.304226 0.000018
O -0.183430 2.575414 -0.000022
C -2.240027 0.555228 -0.000010
O -3.149184 -0.357639 -0.000022
O -1.134965 -2.055818 0.000000
O 3.296700 -0.635377 -0.000025
H 1.389936 -2.349309 0.000018
H 2.220433 1.897964 -0.000010
H -2.617576 1.580686 -0.000012
H 3.908955 0.112201 -0.000041
H -2.726181 -1.251428 -0.000010

[A+H]+, iso5
E = -570.064455
C -2.022566 -0.269600 -0.000032
C -1.078635 -1.299901 0.000021
C 0.397003 -1.062382 0.000289
C 0.877006 0.291402 0.000106
C -0.074838 1.389321 0.000043
C -1.534602 1.045051 0.000013
O 1.120355 -2.060420 0.000073
C 2.243696 0.542801 0.000059
O 3.149280 -0.374206 -0.000256
O 0.197101 2.576915 0.000006
O -3.337968 -0.430155 -0.000137
H -2.228647 1.890229 -0.000042
H -1.377079 -2.352667 0.000006
H 2.626336 1.566373 0.000167
H -3.615804 -1.355590 -0.000177
H 2.722653 -1.265568 -0.000442

[A+H]+, iso6
E = -570.058291
C 2.081796 -0.351542 0.000001
C 1.589372 1.038885 0.000171
C 0.200357 1.316068 0.000328
C -0.756012 0.271265 0.000270
C -0.314549 -1.095165 -0.000063
C 1.069161 -1.424850 -0.000045
O -0.092043 2.603036 -0.000426
C -2.212170 0.527628 0.000315
O -3.022597 -0.380944 -0.000111
O -1.148853 -2.085712 -0.000181
O 3.269437 -0.602858 -0.000001
H 1.387649 -2.467371 -0.000226
H 2.311651 1.855149 0.000184
H -2.082425 -1.733001 0.000020
H -2.578135 1.572498 0.000805
H -1.034024 2.810808 -0.000892

[A+H]+, iso7
E = -570.013647
C -1.623103 -1.014172 -0.000014
C -1.980937 0.349467 0.000163
C -1.033105 1.420592 0.000127
C 0.289655 1.086041 -0.000118
C 0.720413 -0.296154 -0.000040
C -0.282311 -1.336791 -0.000064
O -3.230406 0.668794 0.000189
O 1.295763 1.979304 -0.000502
C 2.055457 -0.600921 0.000192
O 3.140042 0.043146 0.000355
O 0.034547 -2.621461 -0.000214
H -2.383284 -1.790133 -0.000049
H -1.381485 2.451000 0.000002
H 0.987632 -2.776985 -0.000575
H 2.999934 1.018006 0.000820
H 0.981223 2.891472 -0.000294

[B-H], iso1
E = -382.780077
C 0.284508 -1.232146 -0.000548
C 0.997888 0.000171 -0.000271
C 0.284283 1.232355 -0.000512
C -1.083510 1.242582 -0.000564
C -1.846879 -0.000081 0.000402
C -1.083279 -1.242622 -0.000500
C 2.417430 0.000234 0.000040
C 3.631385 -0.000207 0.000875
O -3.089110 -0.000197 0.001084
H -1.649442 -2.173572 -0.001011
H -1.649813 2.173446 -0.001001
H 0.849449 2.163698 -0.000694
H 0.849898 -2.163354 -0.000785
H 4.701830 -0.000344 0.001285

[B-H], iso2
E = -382.734215
C 0.313178 -1.189056 -0.000006
C 1.097949 -0.048629 -0.000105
C 0.380872 1.167048 -0.000015
C -1.006907 1.188081 0.000031
C -1.732601 -0.008535 0.000040
C -1.058805 -1.240627 0.000038
C 2.530510 -0.081361 -0.000095
C 3.740833 -0.090350 -0.000096
O -3.088465 0.083990 0.000115
H -1.603110 -2.186858 0.000100
H -1.550125 2.131305 0.000063
H 0.938201 2.103878 -0.000008
H -3.477870 -0.794563 0.000060
H 4.810451 -0.105108 0.000109

[B-H], iso3
E = -382.719611
6 3.821950 -0.018315 0.000064
6 2.539465 -0.009518 -0.000320
6 1.144544 -0.000295 -0.000001
6 0.419536 1.224316 0.000036
6 -0.957740 1.233977 0.000157
6 -1.657303 0.014647 0.000107
6 -0.969036 -1.211358 0.000134
6 0.410477 -1.217044 0.000060
8 -3.002142 0.085330 -0.000250
1 0.960187 -2.156939 0.000097
1 -1.525496 -2.149953 0.000271
1 -1.522339 2.164593 0.000267
1 0.978863 2.158495 0.000101
1 -3.385428 -0.797297 -0.000159

[B-H], iso4
E = -382.710831
C 0.416921 -1.135482 0.000345
C 1.031583 0.149949 0.000062
C 0.222717 1.316720 0.000250
C -1.143227 1.221035 0.000324
C -1.805905 -0.077246 0.000088
C -0.944217 -1.254610 0.000398
C 2.474560 0.260294 -0.000286

C 3.554077 -0.480658 -0.000469
O -3.043824 -0.175866 -0.000791
H -1.433553 -2.228124 0.000651
H -1.780227 2.104926 0.000497
H 0.703415 2.295469 0.000343
H 2.974349 1.259305 -0.000087
H 1.047558 -2.024662 0.000644

[B-H], iso5
E = -382.665958
C 0.433966 -1.093823 -0.000334
C 1.135304 0.099023 -0.000237
C 0.335057 1.257104 -0.000111
C -1.052331 1.181571 0.000079
C -1.691440 -0.061983 0.000078
C -0.929604 -1.243029 -0.000171
C 2.596083 0.163284 -0.000341
C 3.657631 -0.591917 0.000776
O -3.050009 -0.067054 0.000313
H -1.403919 -2.226161 -0.000232
H -1.659722 2.084698 0.000207
H 0.816076 2.236438 -0.000147
H -3.376796 -0.970782 -0.000231
H 3.116442 1.150858 -0.000536

1A, iso1
E = -569.744237
C 1.924666 -0.298882 0.000090
C 0.966630 -1.318493 -0.000128
C -0.421787 -0.998699 -0.000216
C -0.853892 0.325009 -0.000269
C 0.130262 1.406870 -0.000153
C 1.537901 1.033280 0.000155
O -1.251017 -2.038571 -0.000271
C -2.274023 0.618805 -0.000125
O -3.178295 -0.173820 0.000726
O -0.192683 2.601321 -0.000235
O 3.219169 -0.695746 0.000257
H 2.262093 1.848459 0.000406
H 1.271725 -2.363196 -0.000122
H 3.800727 0.070246 0.000384
H -2.170477 -1.708314 -0.000595

1A, iso2
E = -569.743183
6 1.522146 1.064028 -0.000053
6 0.111594 1.416351 0.000156
6 -0.858354 0.321396 0.000041
6 -0.406487 -0.994048 0.000079
6 0.988857 -1.296544 0.000070
6 1.929696 -0.269603 -0.000032
8 -0.227434 2.607552 0.000061
6 -2.282325 0.589453 -0.000138
8 -3.173287 -0.219049 -0.000103
8 -1.218540 -2.047183 0.000068
8 3.261894 -0.507362 -0.000100
1 1.283250 -2.346349 0.000099
1 2.251916 1.871380 -0.000154
1 3.436356 -1.453079 -0.000069
1 -2.143343 -1.729811 -0.000033

1A, iso3
E = -569.740883
C -2.018691 -0.293469 0.000458
C -1.063930 -1.384423 0.000272
C 0.291668 -1.123752 -0.000299
C 0.742861 0.231408 0.000521
C -0.186765 1.317967 -0.000178
C -1.537301 1.073045 -0.000574
O 1.149987 -2.154659 -0.001252
C 2.162778 0.538994 -0.000008
O 3.100994 -0.201350 0.001200
O 0.252437 2.592010 -0.000076
O -3.244540 -0.536388 0.000574
H -2.260628 1.884895 -0.001273
H -1.437752 -2.406087 0.000433
H 2.064111 -1.833347 -0.000756
H 1.219523 2.599027 -0.003114

1A, iso4
E = -569.778994
6 -2.068396 0.000008 -0.089831
6 -1.306676 -1.256440 0.010976
6 0.034107 -1.263775 0.110221
6 0.780056 -0.000006 0.096214
6 0.034119 1.263770 0.110221
6 -1.306662 1.256451 0.010976
8 0.730841 -2.435751 0.141721
6 2.092325 -0.000005 -0.152226
8 3.232014 -0.000005 -0.342134
8 0.730887 2.435732 0.141706
8 -3.287886 0.000017 -0.208560
1 -1.863586 2.191162 0.015812
1 -1.863609 -2.191145 0.015816
1 1.463508 2.383792 0.763670
1 1.463591 -2.383766 0.763528

3A, iso1
E = -569.744809
C 1.924997 -0.294186 0.000019
C 0.965767 -1.317241 0.000001
C -0.421239 -1.001943 -0.000020
C -0.852389 0.322492 -0.000018
C 0.132087 1.406061 -0.000045
C 1.539873 1.035726 -0.000007
O -1.251054 -2.042915 -0.000099
C -2.273738 0.621637 -0.000028
O -3.177482 -0.170633 0.000139
O -0.197488 2.598470 -0.000032
O 3.218292 -0.695121 0.000057
H 2.263293 1.851415 0.000007
H 1.274600 -2.360927 0.000010
H 3.801978 0.069291 0.000077
H -2.170159 -1.713464 -0.000018

3A, iso2
E = -569.743695
6 1.523782 1.066059 -0.000044
6 0.112939 1.415286 0.000124
6 -0.857179 0.319124 0.000095
6 -0.406119 -0.996665 0.000189
6 0.988169 -1.294892 0.000073
6 1.930303 -0.264995 -0.000049
8 -0.231938 2.604657 0.000113
6 -2.282378 0.591926 -0.000105
8 -3.172729 -0.216314 -0.000270
8 -1.218239 -2.051233 0.000135
8 3.262129 -0.506796 -0.000171
1 1.285667 -2.343935 0.000068
1 2.252421 1.874306 -0.000122
1 3.433834 -1.452941 -0.000163
1 -2.142812 -1.734992 0.000062

3A, iso3
E = -569.742333
6 -2.019860 -0.293985 -0.000002
6 -1.063394 -1.385945 0.000015
6 0.289632 -1.126270 -0.000102
6 0.742909 0.231134 -0.000193
6 -0.186935 1.319069 -0.000089
6 -1.536486 1.074105 -0.000050
8 1.150091 -2.156122 0.000026
6 2.163717 0.542046 -0.000212
8 3.098854 -0.202257 0.000262
8 0.254260 2.592143 0.000152
8 -3.243727 -0.534026 0.000108
1 -2.260467 1.885437 -0.000004
1 -1.438137 -2.407239 0.000016
1 1.221516 2.596879 -0.000322
1 2.063773 -1.833909 -0.000268

A+, iso1

E = -569.503314
6 -1.261568 -1.280155 0.000113
6 0.184733 -1.317371 0.000522
6 0.869260 0.009264 0.000250
6 0.124296 1.218482 0.000225
6 -1.285012 1.170784 -0.000085
6 -1.963007 -0.047829 -0.000221
8 0.823792 -2.360394 0.000217
6 2.229932 -0.040175 -0.000103
8 3.363397 -0.090568 -0.000360
8 0.638916 2.430178 0.000294
8 -3.282129 -0.148640 -0.000643
1 -1.817754 2.121234 -0.000222
1 -1.802225 -2.226086 0.000124
1 -3.727211 0.708424 -0.000900
1 1.603580 2.473824 0.000733

A+, iso2
E = -569.466262
6 1.984288 0.000155 -0.060166
6 1.317178 -1.251211 -0.000731
6 -0.049827 -1.260651 0.081123
6 -0.773714 -0.000177 0.082206
6 -0.050171 1.260543 0.081115
6 1.316861 1.251357 -0.000514
8 -0.658861 -2.443523 0.111339
6 -2.117676 -0.000244 -0.093735
8 -3.246763 0.000042 -0.244605
8 -0.659412 2.443277 0.111271
8 3.268926 0.000383 -0.148119
1 1.875697 2.183225 -0.006058
1 1.876178 -2.182979 -0.006478
1 -1.572582 2.431318 0.418791
1 -1.572039 -2.431625 0.418878

A+, iso3
E = -569.419735
C -2.051752 -0.305011 0.000076
C -1.081806 -1.380442 0.000008
C 0.326040 -1.086480 0.000260
C 0.790965 0.252469 0.000675
C -0.140293 1.309916 0.000241
C -1.554996 1.055655 0.000002
O 1.120834 -2.111919 -0.000227
C 2.226748 0.545171 0.001631
O 3.113967 -0.243762 -0.001031
O 0.205346 2.565599 -0.000571
O -3.271527 -0.541810 -0.000168
H -2.257298 1.889657 -0.000155
H -1.419472 -2.417416 -0.000424
H 2.065707 -1.848426 -0.000047
H 1.172660 2.683652 -0.000755

A+, iso4
E = -569.416978
C 1.987768 -0.006271 0.038013
C 1.311886 -1.241978 0.002341
C -0.121181 -1.360101 -0.058109
C -0.861592 0.002234 -0.021776
C -0.105706 1.356740 -0.058707
C 1.329323 1.224827 0.004022
O -0.745713 -2.392518 -0.127373
C -2.139744 0.009769 0.054607
O -3.350052 0.016208 0.247023
O -0.719036 2.394156 -0.129520
O 3.313578 -0.103156 0.092942
H 1.882913 2.164599 0.012256
H 1.882935 -2.169961 0.010657
H 3.748531 0.757880 0.111674
H -3.909113 0.018638 -0.561512

G+
E = -953.059299
C 2.963528 -1.341702 0.367310
C 2.090475 -0.264878 -0.002081
C 2.694129 0.983084 -0.367569
C 4.055672 1.133902 -0.385885
C 4.887547 0.050371 -0.015745
C 4.320976 -1.193555 0.369423
C 0.663568 -0.433803 -0.013813
C 0.128493 -1.709420 -0.120105
O -1.144246 -2.001273 -0.160001
C -2.100100 -1.005149 -0.081144
C -1.694977 0.336992 0.025200
C -0.302095 0.703238 0.057505
C -2.724772 1.319081 0.105341
C -4.066019 0.934616 0.072914
C -4.399134 -0.414255 -0.036471
C -3.408065 -1.415631 -0.115661
O -2.438174 2.607749 0.211488
O -5.664719 -0.849487 -0.072791
O 0.072479 1.877326 0.164405
O 6.205919 0.125023 -0.000801
H 2.553179 -2.290771 0.706013
H 4.986438 -1.999437 0.672845
H 4.499748 2.083131 -0.687015
H 2.054516 1.814499 -0.645145
H 0.735199 -2.606746 -0.223448
H 6.526491 0.996911 -0.266147
H -4.831622 1.706619 0.136619
H -3.680326 -2.464158 -0.199714
H -6.288010 -0.117444 -0.014846
H -1.461040 2.705344 0.227117

B
E = -383.412817
6 -3.709106 0.014088 -0.000092
6 -2.498089 0.008922 0.000061
6 -1.063335 0.001795 -0.000053
6 -0.352724 -1.209193 0.000031
6 1.034807 -1.221916 0.000001
6 1.738549 -0.015219 -0.000013
6 1.047360 1.198712 0.000027
6 -0.343156 1.203520 0.000039
8 3.097898 -0.085940 -0.000015
1 -0.881460 2.150349 0.000080
1 1.595136 2.142852 0.000035
1 1.589040 -2.159086 0.000053
1 -0.902491 -2.149426 0.000100
1 -4.778782 0.018394 0.000051
1 3.469540 0.800180 -0.000212

B+
E = -383.118854
6 3.671181 -0.014260 -0.000537
6 2.450065 -0.006642 -0.000189
6 1.055051 0.002049 0.000226
6 0.340541 1.244895 0.000379
6 -1.022974 1.251478 0.000273
6 -1.726243 0.009254 -0.000060
6 -1.032725 -1.237416 0.000282
6 0.331805 -1.237388 0.000374
8 -3.034563 0.089770 -0.000627
1 0.889045 -2.172632 0.000537
1 -1.593818 -2.172283 0.000485
1 -1.599656 2.174729 0.000375
1 0.902972 2.176816 0.000540
1 4.745799 -0.020997 -0.000637
1 -3.468048 -0.775612 -0.000775

Figure S7, TS1
E = -952.924980
c -0.364067 2.640033 0.045321
o 1.871609 2.214333 -0.913641
c -0.878822 1.504853 0.126357
c 0.348350 0.751911 1.115771
o -0.126182 0.450415 2.171921
c 1.567122 0.355104 0.508777
c 2.211582 1.069892 -0.592330
c 3.332054 0.427719 -1.254509
c 3.865991 -0.778757 -0.764458
c 3.322417 -1.377784 0.365582

c 2.189004 -0.804375 1.014681
o 1.771068 -1.452654 2.079352
o 4.918835 -1.266550 -1.425737
c -2.039368 0.649881 -0.074405
c -3.325799 1.200759 0.042434
c -4.440106 0.413833 -0.183191
c -1.886386 -0.699463 -0.411745
c -3.000498 -1.490882 -0.643113
c -4.282739 -0.935971 -0.528242
o -5.407176 -1.642607 -0.734102
h -5.209254 -2.555289 -0.966443
h -5.444583 0.823379 -0.096226
h -3.449305 2.246918 0.317936
h -0.892912 -1.136706 -0.511713
h -2.877944 -2.539517 -0.914915
h 0.127104 3.594744 -0.020037
h 1.021598 -0.988978 2.499029
h 3.738097 -2.289667 0.793181
h 5.255196 -2.076994 -1.026878
h 3.794352 0.938097 -2.097852

Figure S7, LM2
E = -952.988115
C 0.236146 0.734541 0.018925
O -2.040671 2.263718 0.020153
C 1.511922 0.556448 0.011235
C -0.736343 -0.424102 0.012449
O -0.278222 -1.573938 0.011763
C -2.146645 -0.131130 0.006765
C -2.719098 1.223554 0.010289
C -4.167665 1.353398 0.002193
C -5.008897 0.234035 -0.008155
C -4.461059 -1.041321 -0.010580
C -3.041909 -1.219448 -0.002902
O -2.640125 -2.468577 -0.005806
O -6.329115 0.473261 -0.015058
C 2.848710 0.364231 0.000328
C 3.559869 0.264817 -1.245747
C 4.908124 0.053349 -1.251659
C 3.568742 0.239880 1.236891
C 4.918566 0.027853 1.226905
C 5.595604 -0.066371 -0.016813
O 6.895553 -0.270199 -0.096733
H 7.312401 -0.344497 0.771323
H 5.476961 -0.028418 -2.175484
H 3.005653 0.358382 -2.178323
H 3.022913 0.314154 2.176265
H 5.475455 -0.070061 2.158782
H -0.206560 1.745783 0.028041
H -1.645952 -2.463477 0.000143
H -5.078175 -1.939344 -0.018295
H -6.836766 -0.345045 -0.022374
H -4.581685 2.360010 0.004628

Figure S7, TS2
E = -952.984156
c 1.771389 -0.794188 0.718520
c 1.944283 0.565614 0.195117
c -0.203001 1.403263 1.329262
c -1.346432 1.032649 0.878078
c 1.007891 1.633521 0.418751
o 0.780928 -1.148409 1.374915
o 1.125635 2.767722 -0.040300
c 2.817211 -1.766687 0.440106
c 3.950993 -1.431731 -0.310519
c 4.096395 -0.144353 -0.811992
c 3.097031 0.847132 -0.561106
o 3.340376 2.027203 -1.084319
o 4.852190 -2.406718 -0.506728
c -2.482756 0.472372 0.407511
c -2.617728 -0.960125 0.456562
c -3.755569 -1.555861 -0.007796
c -3.547692 1.269872 -0.131037
c -4.684408 0.668768 -0.592513
c -4.794089 -0.746247 -0.532519
o -5.865960 -1.384714 -0.960925
h -6.539956 -0.784658 -1.304777
h -3.894900 -2.634733 0.012995
h -1.793257 -1.543294 0.867106
h -3.434659 2.352627 -0.164132
h -5.502754 1.260197 -1.003345
h -0.070395 1.630091 2.390412
h 2.591889 2.629294 -0.848990
h 4.962222 0.154332 -1.402459
h 5.603410 -2.095763 -1.022889
h 2.691933 -2.772751 0.836392

Figure S7, LM3
E = -953.058195
C -0.156149 1.692131 -0.000583
C 1.260069 1.864091 -0.000493
O 0.038982 -0.681541 -0.000048
C -0.732650 0.439224 -0.000269
O 1.861831 2.960633 -0.000654
C 2.018747 0.624693 -0.000123
C 1.379625 -0.592581 0.000058
C 2.111964 -1.793113 0.000337
C 3.547647 -1.738115 0.000437
C 4.224241 -0.536230 0.000246
C 3.465393 0.661006 -0.000002
O 4.033395 1.813395 -0.000135
O 4.128501 -2.935640 0.000748
C -2.154192 0.143576 -0.000158
C -2.614222 -1.190130 -0.001509
C -3.963849 -1.477351 -0.001460
C -3.106674 1.181143 0.001401
C -4.460411 0.903077 0.001576
C -4.898177 -0.430489 0.000124
O -6.193323 -0.773949 0.000163
H -6.762638 0.002678 0.001260
H -4.323131 -2.504437 -0.002609
H -1.903753 -2.012944 -0.002717
H -2.800626 2.225132 0.002535
H -5.184098 1.718304 0.002898
H -0.779522 2.580438 -0.000783
H 3.255032 2.529535 -0.000357
H 5.311036 -0.473968 0.000363
H 5.091368 -2.872659 0.000844
H 1.613072 -2.760848 0.000466

Figure S7, TS3
E = -952.935015
C -0.422746 2.848337 -1.185316
C -0.982742 1.889071 0.928197
O -2.959519 1.426966 -1.052755
C 0.518632 2.072772 -1.102781
O -0.614891 2.687423 1.667387
C -1.483348 0.625602 0.610857
C -2.469926 0.460510 -0.470641
C -2.857260 -0.897529 -0.789921
C -2.315309 -1.994397 -0.089364
C -1.392276 -1.798721 0.931723
C -0.970780 -0.490416 1.291499
O -0.078988 -0.453144 2.267654
O -2.745991 -3.198284 -0.468953
C 1.484434 1.062225 -0.849330
C 1.285261 -0.238756 -1.354316
C 2.171232 -1.254703 -1.048406
C 2.607338 1.322598 -0.043929
C 3.500173 0.307694 0.263658
C 3.282898 -0.985173 -0.235031
O 4.104494 -2.016844 0.024557
H 4.861168 -1.737061 0.549921
H 2.039591 -2.260696 -1.442659
H 0.430739 -0.436922 -2.000750
H 2.778218 2.330262 0.333265
H 4.373894 0.517744 0.881545
H -1.148021 3.596284 -1.443300
H 0.209196 0.436468 2.508132
H -0.960480 -2.631749 1.485184
H -2.362123 -3.907928 0.058833
H -3.596508 -1.050027 -1.574691

Figure S7, LM4
E = -952.944551
C 0.177066 2.841224 1.742635
C 0.812523 1.607293 -1.265597
O 3.177477 1.784258 0.061073
C -0.647436 2.025979 1.392503
O 0.404311 2.539827 -1.765554
C 1.361067 0.481837 -0.725542
C 2.680363 0.672200 -0.067666
C 3.338509 -0.535129 0.385354
C 2.749873 -1.806155 0.190604
C 1.498097 -1.936647 -0.405582
C 0.773957 -0.801619 -0.842436
O -0.404199 -1.049837 -1.361823
O 3.459729 -2.838585 0.632123
C -1.576194 1.033017 0.936597
C -1.730043 -0.179861 1.632418
C -2.617108 -1.148840 1.190021
C -2.345045 1.245070 -0.220108
C -3.231832 0.268221 -0.678296
C -3.375120 -0.931371 0.032236
O -4.217030 -1.915433 -0.341768
H -4.746049 -1.652606 -1.101421
H -2.751641 -2.079935 1.737853
H -1.155498 -0.347529 2.542294
H -2.277450 2.197020 -0.746051
H -3.838168 0.459220 -1.564851
H 0.871540 3.583375 2.081885
H -1.029687 -0.298772 -1.341201
H 1.021008 -2.908209 -0.528690
H 3.023411 -3.684204 0.472538
H 4.308157 -0.441511 0.872384
